# Single-molecule visualization of sequence-specific RNA binding by a designer PPR protein

**DOI:** 10.1093/nar/gkae984

**Published:** 2024-11-12

**Authors:** Nicholas Marzano, Brady Johnston, Bishnu P Paudel, Jason Schmidberger, Slobodan Jergic, Till Böcking, Mark Agostino, Ian Small, Antoine M van Oijen, Charles S Bond

**Affiliations:** University of Wollongong, School of Chemistry and Molecular Bioscience, Molecular Horizons, Northfields Avenue, Wollongong, NSW 2500, Australia; University of Western Australia, School of Molecular Sciences, 35 Stirling Highway, Crawley, WA 6009, Australia; University of Wollongong, School of Chemistry and Molecular Bioscience, Molecular Horizons, Northfields Avenue, Wollongong, NSW 2500, Australia; University of Western Australia, School of Molecular Sciences, 35 Stirling Highway, Crawley, WA 6009, Australia; University of Wollongong, School of Chemistry and Molecular Bioscience, Molecular Horizons, Northfields Avenue, Wollongong, NSW 2500, Australia; University of New South Wales, Department of Molecular Medicine, EMBL Australia Node in Single Molecule Science, Gate 11, Botany St, Sydney, NSW 2052, Australia; Curtin University, Curtin Medical School, Curtin Health Innovation Research Institute, and Curtin Institute for Computation, Kent St, Bentley, WA 6102, Australia; University of Western Australia, School of Molecular Sciences, 35 Stirling Highway, Crawley, WA 6009, Australia; University of Sydney, Faculty of Medicine and Health, G02 Jane Foss Russell Building, Sydney, NSW 2006, Australia; University of Western Australia, School of Molecular Sciences, 35 Stirling Highway, Crawley, WA 6009, Australia

## Abstract

Pentatricopeptide repeat proteins (PPR) are a large family of modular RNA-binding proteins, whereby each module can be modified to bind to a specific ssRNA nucleobase. As such, there is interest in developing ‘designer’ PPRs (dPPRs) for a range of biotechnology applications, including diagnostics or *in vivo* localization of ssRNA species; however, the mechanistic details regarding how PPRs search for and bind to target sequences is unclear. To address this, we determined the structure of a dPPR bound to its target sequence and used two- and three-color single-molecule fluorescence resonance energy transfer to interrogate the mechanism of ssRNA binding to individual dPPRs in real time. We demonstrate that dPPRs are slower to bind longer ssRNA sequences (or could not bind at all) and that this is, in part, due to their propensity to form stable secondary structures that sequester the target sequence from dPPR. Importantly, dPPR binds only to its target sequence (i.e. it does not associate with non-target ssRNA sequences) and does not ‘scan’ longer ssRNA oligonucleotides for the target sequence. The kinetic constraints imposed by random 3D diffusion may explain the long-standing conundrum of why PPR proteins are abundant in organelles, but almost unknown outside them (i.e. in the cytosol and nucleus).

## Graphical Abstract

**Graphical Abstract:**
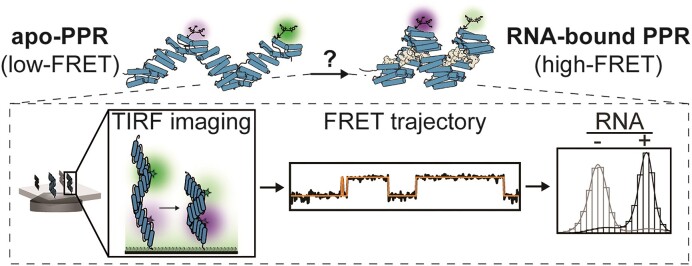

## Introduction

It is increasingly apparent that the plethora of RNA forms (e.g. mRNA, long non-coding RNA, spliceosome components) play complex roles in information transfer and regulation within cells (1). Indeed, the functional output of even a single RNA transcript can vary depending on several factors, such as RNA stability, secondary structure or splicing variations, many of which are regulated by classes of RNA-binding proteins. One of these classes are the pentatricopeptide repeat (PPR) proteins, which are a large family of modular RNA-binding proteins with many roles in RNA stability, processing, splicing and translation (2–7). While prevalent in all eukaryotes, the PPR family is extensively expanded in terrestrial plants and its members are almost uniquely found in organelles (8). PPR proteins bind to specific single-stranded RNA (ssRNA) target sequences; their specificity is ultimately determined by repeating modular motifs of *ca*. 35 amino acids, whereby each consecutive module specifically recognizes a discrete RNA nucleobase. A PPR recognition code (9,10) relates how each nucleotide interacts with two amino acids at the 5th and final (commonly 35th) positions of each motif; amino acid 5 primarily distinguishes purines from pyrimidines and amino acid 35 primarily distinguishes between guanine/uracil or cytosine/adenine bases. The determination of the PPR recognition code has resulted in the emergence of ‘designer’ PPR (dPPR) proteins, which can be rationally modified to target any RNA sequence of interest (11–14). As such, there is significant potential for the use of dPPRs in diagnostics (e.g. RNA detection in biological fluids), as a tool to modulate the expression or silencing of target genes (15–17), or to detect and localize target RNA sequences *in vivo*. However, to be effective, the affinity of a dPPR to its binding site needs to be sufficiently high, yet simultaneously specific to minimize the occurrence of off-target binding.

While the PPR code is a useful tool to help predict PPR binding sites, there is a two-fold degeneracy that often makes the prediction of both PPR binding sites and the sequence specificity of naturally occurring PPR proteins *in vivo* ambiguous (18,19). This degeneracy of the PPR code occurs since multiple amino acid combinations can specify for the same nucleotide base, and conversely, the same amino acid combinations can target a variety of nucleotides. Additionally, not all amino acid combinations bind to nucleotides with equal affinity (20) and the importance of nucleotides for recognition and binding may vary according to their position within the binding site (18,21). Target prediction becomes even more complex in the context of the cellular environment, where PPR protein target sites often contain ‘mismatches’, or are located within long RNA sequences that contain substantial folded secondary structure. While PPR proteins can prevent the formation of RNA secondary structure (7), it remains unclear how transcript length affects the kinetics of PPR binding. Critically, the precise manner by which PPRs search, recognize and bind to their target sequence within much longer transcripts is not known; for example, do PPRs bind to the end of a transcript and ‘scan’ for the target sequence, or do they bind directly to the target (with the ensuing topological issues)? Understanding these fundamental aspects of PPR function may help explain why PPR proteins are predominantly restricted to organelle compartments, which has been a long-standing conundrum in the field. As such, we sought to investigate the precise mechanism(s) by which PPR proteins bind to their target sequences and are challenged by factors present *in vivo* (e.g. mismatches, longer transcripts, etc.) to ultimately improve the prediction of binding sites and the design of synthetic PPR proteins for *in vivo* and diagnostic applications.

To do so, we have designed a synthetic dPPR protein that is a mimic of the model protein *Arabidopsis thaliana* PPR10, such that it is targeted to a 17-nt region of the transcript *atpH* (7), and investigated its binding to a range of RNA variants using a combination of X-ray crystallography, microscale thermophoresis (MST), surface plasmon resonance (SPR) and single-molecule fluorescence approaches. We present the first reported crystal structure of a designer P-class PPR protein (dPPR10) bound to a complex target RNA molecule, in which we observe that dPPR10 becomes conformationally compressed when bound to RNA. Using this information to design and validate a sequence-specific RNA-stimulated FRET-capable protein, we then performed two- and three-color single-molecule fluorescence resonance energy transfer (smFRET) experiments that allow the binding of ssRNA constructs to a single dPPR10 protein to be monitored in real time. We confirm that mismatches toward the center of the binding site, but not the 3′ end, of the cognate sequence are poorly tolerated and that RNA secondary structure affects the accessibility of binding sites to dPPR10. In addition, we demonstrate that dPPR10 does not associate with non-cognate sequences and instead binds directly to the target, suggesting that it has little or no ability to scan RNA transcripts. Together, this implies there would be kinetic constraints on target recognition in complex transcriptomes that would explain why PPR proteins are primarily localized to organelles and not found more widely distributed throughout the cellular milieu.

## Materials and methods

### Materials

#### Nucleic acids

Unmodified or fluorescently labeled RNA and DNA oligonucleotides were purchased from Integrated DNA Technologies (IDT) at the 100 nmol scale. All sequences are provided in Supplementary Figure S1A. Oligonucleotides were resuspended in RNAse-free water to a concentration of 1 mM and stored at −80°C until further use.

#### Generation and confirmation of RNA–DNA duplexes

RNA–primer duplexes were generated by incubating RNA (20 μM) in the absence or presence of increasing molar ratios of DNA primer (1:1–1:2 RNA:primer) in annealing buffer [25 mM Tris (pH = 7.8), 50 mM KCl]. Samples were heated at 95°C for 2 min to resolve any secondary structure or nucleotide dimers and then cooled to 4°C by incremental decreases in temperature (1°C every 38 s, Mastercycler Nexus GX2). DNA primer in the absence of RNA was included as a control. To confirm successful formation of RNA–primer duplexes, the samples were diluted 1:20, loaded onto a 4–20% Mini-PROTEAN TGX gel (Bio-Rad) and run in TAE buffer [40 mM Tris, 20 mM acetic acid and 1 mM ethylenediaminetetraacetic acid (EDTA)] at 100 V for 50 min. The gel was then treated in fixing buffer [10% (*v/v*) methanol, 7% (*v/v*) acetic acid and 5% (*v/v*) glycerol] for 15 min, washed 3× in milli-Q water and then stained with 1× SYBR-Gold in TAE buffer for 15 min. The gel was then imaged using a Typhoon imager as per the manufacturer’s instructions. Samples containing complete RNA–primer duplexes were then stored at −80°C until use.

#### Protein expression, purification and chemical labeling

The design of the dPPR10 construct followed the methodology put forward in Gully *et al.* (2015) (11), which involved repeating a consensus 35-amino-acid repeat sequence 17 times, changing residues 5 and 35 of the motif to target it to each base of the target RNA. The final amino acid sequence of purified, cleaved dPPR10 protein is GAMGND-(VVTY[NT]TLIDGLAKAGRLEEALQLFQEMKEKGVKP [DNS])_17_-VVTNNTLKDGASKAG, where the bracketed motif is repeated, and the square-bracketed underlined residues that vary (Supplementary Figure S2). The sequence of the variant protein dPPR10-C2 is identical to dPPR10 except for the substitution of residues Gln96 and Gln376 to cysteine, and the inclusion of an N-terminal Avi-tag sequence for biotinylation (Supplementary Figure S2). The complete protein sequences for dPPR10 and dPPR10-C2 are given in Supplementary Table S1. Genes were codon-optimized, flanked with NcoI and EcoRI restriction endonuclease sites, synthesized and delivered in a pUC57 vector by GenScript. The insert was subsequently subcloned into pETM20 (a gift from Gunter Stier, EMBL Protein Core Facility, Heidelberg, Germany; https://www.embl.de) for protein overexpression and purification. Transformed colonies were plated on Lysogeny Broth (LB) agar plates supplemented with ampicillin (100 μg/ml) for selection and maintenance of the pETM20 plasmid.

The dPPR10 protein was overexpressed in Rosetta 2™ (DE3) *Escherichia coli*, following the method of Gully *et al.* (2015) (11) with minor modifications. LB medium (0.5 l) was innoculated with transformed cells and incubated with shaking at 180 rpm at 37°C until the culture reached an optical density of 0.6. Expression was induced with 0.5 mM isopropyl ß-D-1-thiogalactopyranoside (IPTG), then shaken at 16°C for 18 h.

For biotin-mediated immobilization of dPPR10-C2, the Avi-tag/BirA coexpression method was used (22). Vector pETM20-avi-dPPR10-C2 was co-expressed in BL21(DE3) with pCY216, which encodes the biotin ligase BirA under control of an arabinose promoter (a gift of Steven Polyak, University of Adelaide). When cells, cultured in LB medium supplemented with a final concentration of 0.2 mg/ml biotin, reached an optical density of 0.6, dPPR10-C2 and BirA were both induced upon addition of 0.5 mM IPTG and 0.05% (*w/v*) L-arabinose and incubated overnight at 16°C at 180 rpm for 18 h.

Cells were harvested by centrifugation at 4000 x g for 10 min with pellets frozen and stored at −20°C. Pellets were resuspended in 50 mM Tris-HCl (pH = 8.0), 500 mM KCl, 10% (*v/v*) glycerol (lysis buffer) with a mini cOmplete protease inhibitor (EDTA free) tablet (Roche) and 0.5 μl benzonase 250 U/μl (Sigma Aldrich). Bacteria were lysed under high pressure using an Emulsiflex C5 Homogeniser (Avestin). The insoluble fraction was pelleted by centrifugation at 24 000 g for 30 min at 4°C. The supernatant containing soluble proteins was loaded on to a 5 ml HisTrap Column (GE Healthcare) at 2.5 ml/min and washed at 2.5 ml/min with 10% elution buffer (lysis buffer + 500 mM imidazole). Protein was eluted from the column using a 10–100% gradient of elution buffer over eight column volumes. Protein-containing fractions were pooled and dialysed overnight into lysis buffer in the presence of 4 mg TEV protease and dithiothreitol (DTT) (1 mM). The cleaved protein was washed through a 5 ml HisTrap column to remove any remaining His-tagged protein impurities. SDS-PAGE evaluated protein purity (Mini-PROTEAN TGX Stain-Free Gels, 4–20% 15-well comb, Bio-Rad). Pure protein fractions were typically concentrated to 12–14 mg/ml using centrifugal concentration [Amicon™ Ultra – 30 kDa molecular weight cut-off (MWCO), Merck Millipore] before being snap-frozen in liquid nitrogen and stored at −80°C.

#### Protein labeling with fluorescent dyes

dPPR10-C2 was fluorescently labeled with a Cy3 and Alexa Fluor 647 (AF647) FRET-pair as described previously (23) with minor modifications. Briefly, dPPR10-C2 (∼2 mg/ml) was incubated in the presence of 5 mM Tris(2-carboxyethyl)phosphine and placed on a rotator for 1 h at 4°C. dPPR10 was then incubated in the presence of a 4-fold and 6-fold excess of pre-mixed Cy3 donor and AF647 acceptor fluorophores, respectively, and placed on a rotator overnight at 4°C. Following the coupling reaction, excess dye was removed by gel filtration chromatography using a 7K MWCO Zeba Spin Desalting column (Thermo Fisher Scientific, USA) equilibrated in 50 mM Tris (pH = 7.5) supplemented with 20% (*v/v*) glycerol. The concentration and degree of labeling were calculated by ultraviolet absorbance.

### Structure determination and biophysical characterization of dPPR10

#### Analytical size exclusion chromatography

Protein, RNA and protein–RNA samples were made up to total volume of 25 μl in 50 mM Tris-HCL (pH = 8.0) and 100 mM KCl. Protein was diluted to 1 mg/ml (15 μM), with RNA added at a final molar ratio of 1:1.2. A volume of 12.5 μl of sample was injected onto a Superdex 200 increase 5/150 column (Cytiva), at a flow rate of 0.3 ml/min. Absorbance was measured at 280 and 260 nm using a Biologic Quadtech UV/VIS detector (Bio-Rad).

#### Crystallographic structure determination

Concentrated (12.4 mg/ml) dPPR10 was incubated with its target ssRNA sequence (denoted as RNA^cognat;^, Supplementary Figure S1A) at a 1:1.2 molar ratio and applied to several commercially available 96-well crystallization screens using an Art Robbins Phoenix, SBS-format Intelliplates and drops formed from 100 nl protein and 100 nl reservoir. Diffraction-quality crystals grew in 100 mM magnesium acetate, 50 mM MES (pH 5.6) and 20% 2-methyl-2,4-pentanediol through sitting drop vapor diffusion.

Single crystals with dimensions of *ca*. 100 μm were harvested directly from the 96 well plate in a nylon loop (Hampton Research), flash frozen in liquid nitrogen and screened for diffraction at the MX2 beamline of the Australian Synchrotron (24,25). A complete dataset to 2.0 Å resolution was collected on a Dectris Eiger 16M detector, at 100 K, with 0.1° $\phi$ rotations, 180° wedge collected over 18 s with 60% beam attenuation and a crystal-to-detector distance of 315 mm.

Diffraction data were indexed, merged and integrated using XDS (26). Subsequent processing used the CCP4 suite (27). Scaling and space group identification was performed using AIMLESS (28), and the structure was solved by molecular replacement using PHENIX (29) with a search model modified from PDB 5I9F using a single protein chain containing eight PPR motifs (12). Model building was completed using iterative cycles of graphical building in COOT (30) and refinement in REFMAC (31) cycles. The refined structure was deposited at the Protein Data Bank (32) with code 6EEN.

#### SPR measurements

Binding studies were performed in SPR buffer [30 mM Tris (pH = 7.6), 40 mM KCl, 5 mM MgCl_2_, 0.5 mM DTT, 0.25 mM EDTA and 0.01% (*v/v*) P20 surfactant]. Temperature was kept constant at 20°C. Biotinylated dPPR10-C2 was immobilized onto the surface of streptavidin-coated (SA) SPR chip by injecting 50 nM dPPR10-C2 at a flow rate of 5 μl/min for 750 s. This yielded *ca*. 1650 response units (RUs) of dPPR10 on the surface. Successive injections of 1 M MgCl_2_ and subsequently 2/3/4 M MgCl_2_ resulted in the loss of some non-specifically bound dPPR10s from the surface, leaving ∼1300–1500 RUs of immobilized dPPR10. Given the molecular weight of protein (∼70 kDa) and RNA species (∼5 kDa), in the case of a 1:1 binding, the response is expected to increase by ∼100 RUs upon saturation with RNA (R_max_). The association of RNA from solution to immobilized dPPR10-C2 was monitored following injection of RNA (1 μM) onto the chip at a flow rate of 40 μl/min for 300 s, while its removal is followed by monitoring dissociation over 1000 s.

#### Solution FRET assay

Fluorescently labeled dPPR10-C2 was thawed and diluted to a final concentration of 5 nM in 100 mM Tris-HCl (pH = 8.0), 100 mM KCl. RNA^cognate^ or a ssRNA sequence that was not predicted to bind to dPPR10-C2 based on the PPR code (denoted as RNA^anti-cognate^; Supplementary Figure S1A) was diluted in a 12-point concentration series from 1000 nM to 0.01 nM. A variable mode scanner (Amersham Typhoon™, GE Healthcare) was used to measure fluorescence in 96-well microplate, μClear^®^, Black, non-binding (Greiner Bio-One) with images acquired at a 100 μm per pixel resolution. Fluorescence was recorded on two photon-multiplier tubes with voltages set at 600 V. FRET was calculated from the donor (excitation at 532 nm, emission between 560–580 nm) and transfer (excitation at 532 nm, emission between 655–685 nm) channels, following Equation 1.

#### Microscale thermophoresis

MST experiments used a Monolith NT 115 Series (NanoTemper Technologies) using 20% LED and 40% infrared-laser power for all experiments. Laser on and off times were 30 and 5 s, respectively. The dilution series (typically 1:1) was prepared by serially diluting a sample of dPPR10 [10 μg/ml (i.e. 150 nM)] in 100 mM Tris-HCl (pH = 8.0) and 100 mM KCl, which was then mixed with 5′ 6-FAM labeled RNA oligonucleotides (IDT; RNA^cognate^ or RNA^anti-cognate^) to a final RNA concentration of 50 nM. Protein concentration limits were adjusted based on initial experiments to create a complete binding curve. Data was processed and binding curves calculated using MO Affnity Analysis 2.2.7.6056 (NanoTemper).

### Total internal reflection fluorescence microscopy

#### Microscope setup

Samples were imaged using a custom-built total internal reflection fluorescence (TIRF) microcopy system constructed using an inverted optical microscope (Nikon Eclipse TI) that was coupled to an electron-multiplied charge-coupled device (EMCDD) camera (Andor iXon Life 897, Oxford Instruments, UK). The camera was integrated to operate in an objective-type TIRF setup with diode-pumped solid-state lasers (200 mW Sapphire; Coherent, USA or Stradus 637–140, Vortran Laser Technology, USA) emitting circularly polarized laser radiation of either 488-, 532- or 647-nm continuous wavelength. The laser excitation light was reflected by a dichroic mirror (ZT405/488/532/640; Semrock, USA) and directed through an oil-immersion objective lens (CFI Apochromate TIRF Series 60x objective lens, numerical aperture = 1.49) and onto the sample. Total internal reflection was achieved by directing the incident ray onto the sample at the critical angle (θ_c_) of ∼67° for a glass/water interface. The evanescent light field generated selectively excites the surface-immobilized fluorophores, with the fluorescence emission passing through the same objective lens and filtered by the same dichroic mirror. For two-color experiments, the emission was then split using a T635lpxr-UF2 dichroic (Chroma, USA), passed through ET690/50m and ET595/50m (Chroma, USA) cleanup filters and the final fluorescent image projected onto the EMCDD camera. For three-color experiments, the emission was split using T635lpxr-UF2 and T550lpxr-UF2 dichroics, passed through ET690/50m, ET595/50m and ET525/50 cleanup filters and the final fluorescent image projected onto the EMCDD camera. The camera was running in frame transfer mode at 5Hz, with an electron multiplication gain of 700, operating at −70°C with a pixel distance of 160 nm (in sample space).

#### Coverslip preparation and flow cell assembly

Coverslips were functionalized with neutravidin as previously described (33), with minor modifications. Briefly, 24 × 24 mm glass coverslips were cleaned by alternatively sonicating in 100% ethanol and 5 M KOH for a total of 2 h before aminosilanization in 2% (*v/v*) 3-aminopropyl trimethoxysilane (Alfa Aesar, USA) for 15 min. NHS-ester methoxy-polyethylene glycol, molecular weight 5 kDa (mPEG) and biotinylated-mPEG (bPEG; LaysanBio, USA), at a 20:1 (*w/w*) ratio, was dissolved in 50 mM 4-morpholinepropanesulfonic acid (pH = 7.4) buffer and sandwiched between two activated coverslips for a minimum of 4 h for initial passivation in a custom-made humidity chamber. PEGylated coverslips were then rinsed with milli-Q and PEGylated again as described above for overnight (∼20 h) passivation. PEGylated coverslips were rinsed with milli-Q water, dried under nitrogen gas, and stored at −20°C until required. Prior to use, neutravidin (0.2 mg/ml; BioLabs, USA) in milli-Q was incubated on the passivated coverslip for 10 min to bind to the bPEG. Neutravidin functionalized coverslips were then rinsed with milli-Q, dried under nitrogen gas and bonded to a polydimethylsiloxane flow cell for use in single-molecule experiments. Finally, to reduce the non-specific binding of proteins to the coverslip surface, each channel in the microfluidic setup was incubated in the presence of 2% (*v/v*) Tween-20 for 30 min as previously described (34) and then washed with imaging buffer.

### smFRET experiments and analysis

#### Surface immobilization of labeled proteins and acquisition of smFRET data

For smFRET experiments, fluorescently labeled dPPR10-C2 was specifically immobilized to a neutravidin-functionalized and Tween-20-coated coverslip. To do so, labeled protein (∼50 pM final concentration) was diluted in imaging buffer [50 mM Tris (pH = 8.0), 250 mM KCl and 6 mM 6-hydroxy-2,5,7,8-tetramethylchroman-2-carboxylic acid (Trolox)] and incubated in the flow cell for 5 min. These conditions would typically give rise to ∼75–100 FRET-competent molecules per 100 × 100 μm. Unbound proteins were then removed from the flow cell by flowing through imaging buffer.

For two-color experiments, data was acquired using the TIRF microscope setup previously described following sample illumination using a 532-nm solid state laser with excitation intensity of 2.6 W/cm^2^ and the fluorescence of donor and acceptor fluorophores was measured every 200 ms at multiple fields of view. For three-color experiments, the sample was alternatively illuminated using a 532- and 488-nm solid state for 200 ms per frame and the fluorescence of all dyes measured. An oxygen scavenging system consisting of 5 mM protocatechuic acid and 50 nM protocatechuate-3,4-dioxygenase was included in all buffers prior to and during image acquisition to minimize photobleaching and fluorophore blinking.

To monitor dPPR10-C2 conformational changes upon incubation with ssRNA constructs, a combination of live-flow and steady state smFRET experiments were performed. For live-flow conditions, imaging was initiated and ssRNA (1 μM) was injected into the microfludic flow cell using a syringe pump (300 μl, NE-1000, ProSense B.V) after 10 s; imaging continued for 6 min to observe subsequent changes in FRET efficiency. Multiple fields of view were subsequently imaged for 6 min each in the presence of the incubated ssRNA and the data collated (i.e. steady-state conditions, minimum of 30 min). Three-color experiments were performed as described above, with the exception that the concentration of fluorescently labeled ssRNA (20 nM) was reduced to prevent fluorescence background.

#### Molecule selection and FRET calculations

Single-molecule time trajectories were analyzed in MATLAB using the MASH-FRET user interface (version 1.2.2, accessible at https://rna-fretools.github.io/MASH-FRET/) (35). The approximate FRET value is measured as the ratio between the acceptor fluorescence intensity (*I*_Acceptor_) and the sum of both donor (*I*_Donor_) and acceptor fluorescence intensities after correcting for crosstalk between donor and acceptor channels. The formula for calculating the FRET efficiency is given by Equation 1, whereby the corrected acceptor intensity (denoted as *CI*_Acceptor_) is equal to *I*_Acceptor_*–* (g * *I*_Donor_) and g is the crosstalk correction constant. g is calculated as the ratio of fluorescence measured in the acceptor and donor detection channels following direct excitation of a protein labeled with a single donor fluorophore.


(1) \begin{equation*}{\mathrm{FRET\ = \ }}\frac{{{\mathrm{C}}{{{\mathrm{I}}}_{{\mathrm{Acceptor}}}}}}{{{\mathrm{C}}{{{\mathrm{I}}}_{{\mathrm{Acceptor}}}}{\mathrm{ + \ }}{{{\mathrm{I}}}_{{\mathrm{Donor}}}}}}\end{equation*}

Briefly, donor and acceptor fluorescence channels were aligned following a local weighted mean transformation of images of TetraSpeck fluorescence beads and donor and acceptor fluorescence spots co-localized to identify FRET pairs. Molecules that displayed clear donor and/or acceptor photobleaching events or demonstrated anti-correlated changes in donor and acceptor fluorescence intensity were selected for subsequent analysis. The number of photobleaching events observed was used to determine the number of fluorophores present; only molecules in which a single donor and acceptor photobleaching event was observed were used for further analysis.

#### Trace processing and hidden Markov Model fitting

Selected molecules were denoised in MASH-FRET using the NL filter, which has been described previously (36), to accurately identify and quantify transitions between different FRET states during downstream processing. Parameter values were as follows; exponent factor for predictor weight, 5: running average window size, 1: factor for predictor average window sizes, 2. Data were truncated to only include FRET values acquired before donor or acceptor photobleaching. FRET efficiency data were exported to the state finding algorithm vbFRET (version vbFRET_nov12, https://sourceforge.net/projects/vbFRET/) and trajectories fit to a hidden Markov Model (HMM) to identify discrete FRET states, their residence times and the transition distributions between them. Default vbFRET settings were employed to fit data to the HMM, with the exception that the *mu* and *beta* hyperparameters were changed to 1.5 and 0.5, respectively, to prevent over-fitting. For three-color experiments, the FRET traces were fit with an HMM using custom-written scripts in Python.

#### Kinetic analysis of HMM fits

The HMM fits of individual FRET trajectories were further analyzed to extract key kinetic information arising from changes in PPR conformation. To investigate transitions of interest, each transition (as determined from the HMM analysis) was sorted into different directional classes denoted generally as T_Before-After_, whereby ‘before’ refers to *F*_Before_ and ‘after’ refers to *F*_After_. For simplicity, FRET data were binned according to whether *F*_Before_ or *F*_After_ was >0.5 (high) or <0.5 (low), unless otherwise indicated, and thus two different transition classes are possible: T_high-low_ and T_low-high_. The residence time (defined as the time that a molecule resides at *F*_Before_ prior to transition to *F*_After_) for each transition within a transition class was calculated and presented cumulative histograms. Since the FRET data could be well described as a two-state system, the cumulative residence times (determined as the period of time that the HMM fit resides at low-FRET states prior to a T_low-high_ transition, and vice-versa for T_high-low_ transitions) are presented. Since it is not possible to determine for how long a particular FRET state would have existed if not truncated due to photobleaching, the last measured FRET state was deleted and thus excluded from residence time calculations. Since data were smoothed during denoising, residence times shorter than that given by Equation 2 were not considered for further analysis. *F* is the imaging frame rate in milliseconds and *N_FA_* is the number of frames that were averaged during trace denoising.


(2) \begin{equation*}{\rm Residence} \ {\rm tim}{{{\rm e}}_{{\rm Delete}}}\ = \ 2\ \cdot\ F\ \cdot\ {{N}_{FA}}. \end{equation*}

The rate of ssRNA binding-and-release events was determined by calculating the number of transitions to FRET states below (i.e. release event) or above (i.e. binding event) the FRET threshold set for ssRNA binding (0.5 FRET) for each molecule. From here, the minimum number of binding or release events was divided by the imaging lifetime of that molecule (i.e. time until photobleaching) to determine the binding-and-release rate. Finally, to determine whether there are changes in FRET immediately prior to ssRNA binding, all T_low-high_ transitions were identified (which are indicative of ssRNA binding), filtered such that only transitions with a residence time > 10 s were selected, and the FRET efficiency 10 s prior to and after each T_low-high_ was plotted. The FRET efficiency of all datapoints for each molecule was collated and are presented as FRET efficiency histograms.

### Statistical analysis

To determine how different ssRNA constructs affect the lifetime of certain FRET states, the residence times for each transition class were statistically analyzed using a one-way analysis of variance (ANOVA) with a Tukey’s multiple comparisons *post-hoc* test, with *P* ≤ 0.05 determined to be statistically significant. All data analysis and presentation were performed using custom-written scripts on Python software or using GraphPad Prism 9 (GraphPad Software Inc; San Diego, USA). All original code for the analysis and visualization of smFRET data has been deposited at Zenodo at the following DOI: 10.5281/zenodo.10989968.

## Results

### Designer PPR proteins undergo a predictable, uniform conformational change upon binding RNA

**Figure 1.**
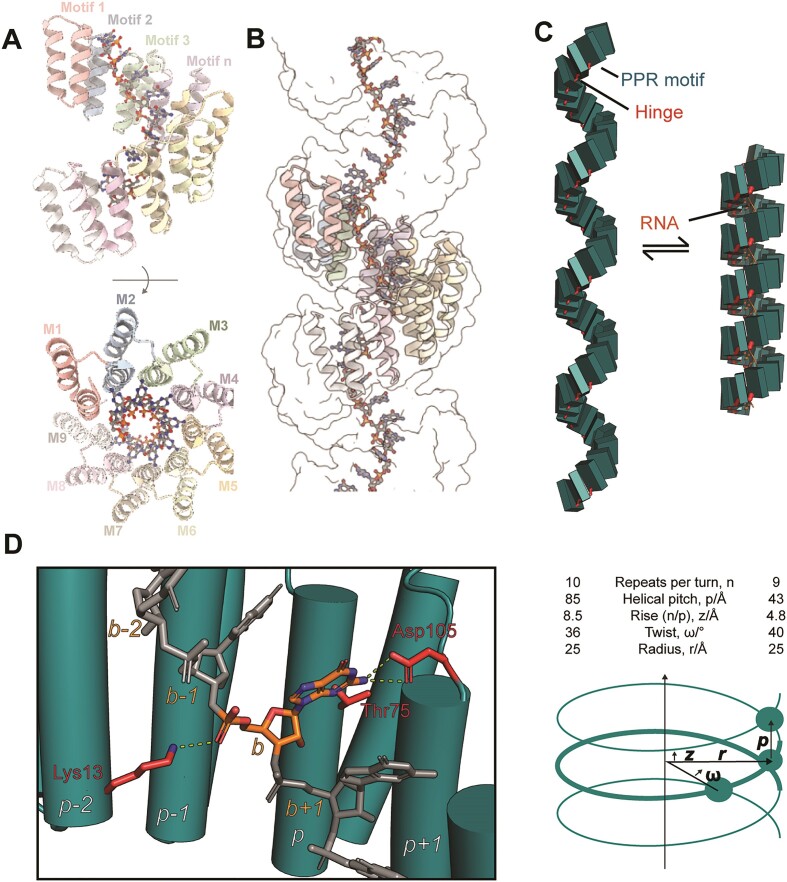
Structural parameters of conformational change of dPPR10 on binding its target RNA. (**A**) Cartoon representation of orthogonal views of the dPPR10 crystal unit cell contents. The individual PPR motifs are denoted in text. (**B**) Neighboring unit cells (shown as surface representation) result in an extended idealized superhelical protein:RNA complex structure. (**C**) Comparison of dPPR (PDB 4OZS) and dPPR10:RNA (PDB 6EEN) structural parameters reveals a dramatic, uniform conformational change on RNA binding (boxes represent individual PPR motifs). (**D**) Each nucleotide interacts with amino acids (e.g. Lys13 from *p-2*, Thr75 and Asp105 from motif *p* for nucleobase *b*) from three adjacent PPR motifs that act to stabilize the bound state. Such interactions likely do not contribute to PPR-RNA specificity. Note that Asp105 is the last residue of motif *p* and directly precedes the start of motif *p + 1*. Figure prepared using PYMOL (The PyMOL Molecular Graphics System, Version 2.0 Schrödinger, LLC.), PDB-MODE (40) and Blender [Community, B. O. (2018). Blender – a 3D modeling and rendering package. Stichting Blender Foundation, Amsterdam. Retrieved from http://www.blender.org] with Molecular Nodes plugin (Johnston, 2022 ‘MolecularNodes’. Retrieved from https://bradyajohnston.github.io/MolecularNodes/).

The complex of dPPR10 bound to its cognate target (RNA^cognate^; including the 17 nt fragment of *atpH* RNA) was readily crystallized in spacegroup *P1* (unit cell: 43.35 Å, 51.65 Å, 51.97 Å, 118.12°, 97.21°, 96.00°), yielding a structure to 2.0 Å resolution (*R*= 0.183, *R*_free_ = 0.243, Figure 1A and Supplementary Table S2). While the protein–RNA complex contains 17 PPR repeats, the crystal structure reflects a continuous protein–RNA superhelical complex (Supplementary Figure S3A) with nine repeats per superhelical turn (Figure 1A). Helical disorder observed in this structure is of the same form reported previously for tetratricopeptide repeat and PPR proteins (11,37), resulting in a structure with excellent electron density and geometry for an extensive protein–RNA molecule that spans the crystal (Figure 1B and Supplementary Figure S3A). Microheterogeneity at the nucleobases and the base-recognizing amino acid sidechains in positions 5 and 35 of each repeat results in averaged electron density for these regions (Supplementary Figure S3B).

Both the dPPR10:*atpH* structure and the structure of dPPR apo-protein (11) (PDB entry 4OZS), which effectively differs from dPPR10 only in the base-interacting amino acids 5 and 35, reveal an idealized model of the PPR α-solenoid superhelix that allows objective measurement and comparison of superhelical parameters (Figure 1C). The dPPR10:*atpH* superhelix is substantially contracted in comparison with the apo-protein (Figure 1C), as expected based on previous reports (11,12,38). The superhelical pitch, which can be measured directly from the lattice parameters, is almost halved to 43 Å (*cf*. 85 Å for apo-protein), and is overwound, with nine repeats per helical turn (*cf*. ten). Using the parameters defined previously (39), this corresponds to a rise of 4.8 Å (*cf*. 8.5 Å), a twist of 40° (*cf*. 36 Å) and a consistent superhelical radius of ∼25 Å.

To gain a better understanding of the mechanism of conformational change on RNA binding, we performed a detailed geometric analysis. Initial inspection showed that the local structural changes are surprisingly subtle relative to the substantial compaction of the superhelix. The program DYNDOM (41) reveals that individual PPR repeats can be approximated as rigid bodies (root-mean-square deviation [RMSD] 0.4–0.6 Å), which rotate with respect to each other around a contiguous hinge comprised of residues 33–35 at the junction of adjacent motifs (Figure 1C and Supplementary Figure S3C). Rotation of 12° ± 4° and translation of up to 1.2 Å about this hinge allows interconversion between the extended and compact structures. Close investigation of backbone geometry (Supplementary Figure S3D) shows that apart from the flexible hinges, the individual motifs are relatively rigid. However, notable torsion occurs at residues 13, 16, 17, 27 and 32–35. Of these residues, 32–35 are involved in the hinge, while Met27 is a highly conserved residue which interacts directly with the hinge residues. Residues 16 and 17 comprise the two-residue linker between helices within a PPR repeat and may thus be expected to absorb some torsional strain. Experimentally, the contraction of dPPR10 upon binding RNA^cognate^ is readily observed in size-exclusion chromatography, where the complex runs slower than the apo-protein despite having a larger mass, due to its compact hydrodynamic profile (Supplementary Figure S4A).

Notably, Lys13 is a highly conserved RNA-interacting residue, which may have importance linking RNA binding to the change in orientation of PPR repeats. Any given RNA nucleotide binds a span of three consecutive PPR repeats (Figure 1D), such that (i) nucleobase *b* is recognized by sidechains of residues 5 and 35 from PPR motif *p* and, (ii) the 5′-phosphate group of nucleobase *b* is bound by a salt-bridge from Lys13 from PPR motif *p*-2. As the mean distance between adjacent Lys13-Nζ atoms reduces from 9.2 ± 2.7 Å to 6.7 ± 0.2 Å on binding to RNA, it is likely that coulombic repulsion between these lysine residues contributes to the more extended apoprotein structure (Supplementary Figure S3E). Taken together, this implies that the sequential binding of nucleotides proceeds via conformational changes to overlapping three-motif segments of the PPR protein.

### Binding of RNA by designer PPR proteins can be detected by conformational change-induced increases in intramolecular FRET

**Figure 2.**
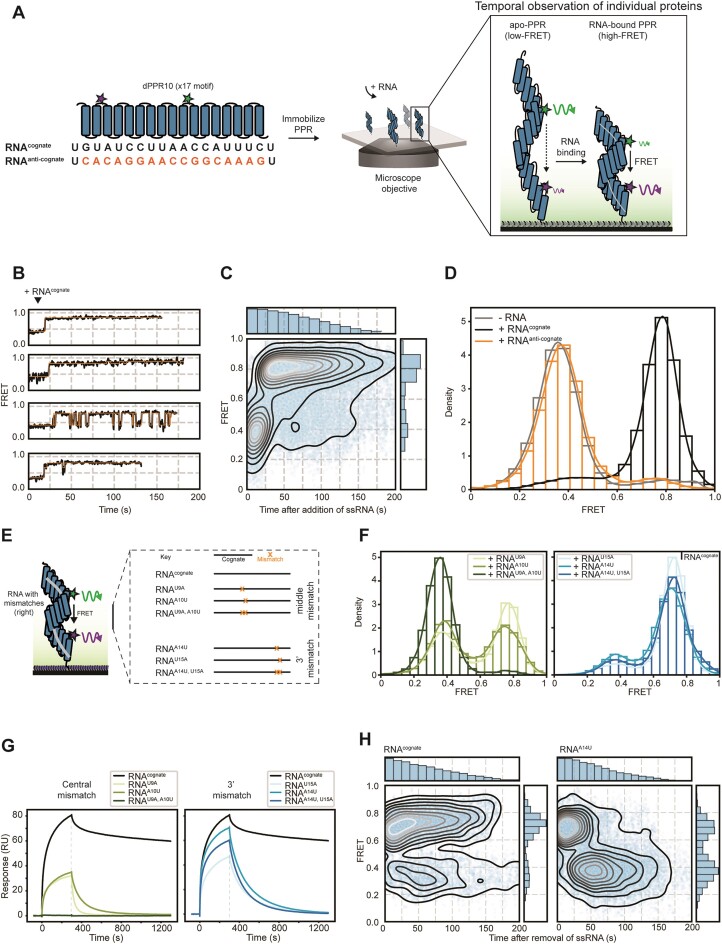
smFRET can be used to monitor dPPR10 conformational changes upon association or dissociation of target and mismatched ssRNA oligonucleotides. (**A**) Schematic of the dPPR10 protein and imaging setup used for smFRET experiments. The architecture of dPPR10 shows the alignment of each motif with its corresponding nucleobase in the cognate sequence (RNA^cognate^) and approximate location of attached fluorophores used for smFRET (*left*). Note that the first and last nucleotide of RNA^cognate^ does not pair with a PPR motif. The Cy3/AF647-labeled dPPR10 protein is immobilized to a coverslip surface and the fluorescence of both dyes are measured and used to determine the FRET efficiency of protein in the absence or presence of ssRNA (*right*). (**B**) Representative FRET trajectories of dPPR10 upon injection of RNA^cognate^ (1 μM) after 10 s (indicated above the traces). (**C**) A heatmap of the FRET intensity over time following the injection of RNA^cognate^ as described in panel (D). Histograms show the decrease in data density due to photobleaching over time (*top*) and the collated FRET efficiency (*right*). Data is collated from 168 individual molecules. (**D**) FRET histogram of dPPR10 incubated in the absence or presence of the indicated ssRNA oligonucleotides (1 μM). Data is collated from at least 114 individual molecules. (**E**) Schematic showing the position of different mismatches introduced into RNA^cognate^ for subsequent smFRET experiments. The key denotes the sequences that are identical (*black*) or different (*denoted by 'X'*) to RNA^cognate^. (**F**) FRET histogram of dPPR10 incubated in the absence or presence of the indicated ssRNA oligonucleotides (1 μM). ssRNA sequences containing mismatches in the dPPR10 region probed by FRET (*left*) and those at the 3′ end (*right*) is indicated. Data is collated from at least 146 individual molecules. (**G**) SPR traces of ssRNA constructs (1 μM) binding to immobilized dPPR10. Association phase of ssRNA to dPPR10 is initiated at t = 0 and proceeds for 300 s. Dissociation curves are generated following removal of ssRNA from solution. Data for RNA^U9A^, RNA^A10U^ and RNA^U9A, A10U^ (*left*) or RNA^U15A^, RNA^A14U^ and RNA^A14U, U15A^ (*right*) are shown. (**H**) Heatmaps of the FRET intensity over time following the removal of ssRNA (at 10 s) from dPPR10 molecules previously incubated with and bound to RNA^cognate^ (*left*) or RNA^A14U^ (*right*). Data is collated from at least 43 individual molecules.

Using our idealized RNA-bound and protein-only structures, we comprehensively evaluated the geometric relationship of pairs of residues to select which residues if suitably labeled would span the Förster critical transfer distance for commonly used FRET pairs. We identified Gln22 of PPR motifs 8 repeats (280 residues) apart as being potentially suitable for this purpose; these residues are polar, located on the external face of the superhelix, and have an inter-residue distance of 71 Å in the absence of RNA, and 40 Å in its presence, which lie on either side of the Förster critical transfer distance (54 Å) for a Cy3/Alexa Fluor 647 (AF647) FRET pair (Figure 2A). Substitution of these residues in repeats 3 and 11 to cysteine (Supplementary Figure S2) and optimization of a dual-labeling reaction with maleimide-functionalized dyes resulted in a sample in which ∼50% of the protein is labeled with both dyes. In a plate based fluoresence assay, dPPR10 showed concentration-dependent changes in acceptor fluorescence in the presence of the RNA^cognate^ oligonucleotide commensurate with a dissociation constant of 1.2 nM (Supplementary Figure S4B and C), confirming the suitability of replacing Gln22 residues with dye-attaching Cys residues. This binding affinity compares favourably to the 4 nM dissociation constant determined by MST for unsubstituted, unlabeled dPPR10 protein binding to FITC-labeled RNA^cognate^ (Supplementary Figure S4D). Both FRET and MST ensemble assays showed that dPPR10 does not bind to the non-cognate RNA^anti-cognate^.

### The binding of cognate or mismatched ssRNA to individual dPPR10 proteins can be monitored temporally using smFRET

While methods that analyze large populations of molecules in bulk as time- or ensemble-averages (such as crystallography, spectroscopy, thermophoresis, SPR) can be used to reveal important aspects of structure, thermodynamics and kinetics, single-molecule methods have the power to interrogate mechanistic details in ways that other methods simply cannot. We therefore sought to take advantage of the significant conformational rearrangements of dPPR10 upon binding to ssRNA (Figure 1C), and its demonstrated capability as a FRET-based sensor (Supplementary Figure S4), by developing a smFRET assay that could be used to monitor dynamic changes in protein conformation in the presence of various ssRNA oligonucleotides. To do so, we generated an enzymatically biotinylated dPPR10 protein (i.e. dPPR10-C2, referred to as dPPR10 henceforth for simplicity) labeled with Cy3 and AF647 that could be specifically immobilized to a coverslip surface via streptavidin-biotin interactions and imaged in the absence or presence of a selection of ssRNA variants (Figure 2A). Using microfluidics, we initiated the binding of ssRNA to dPPR10 by injecting its target ssRNA oligonucleotide (RNA^cognate^) after 10 s of imaging (Figure 2B and C). Initially, in the absence of ssRNA, dPPR10 adopts a stable, low-FRET (∼0.4) conformation that is consistent with the expanded α-solenoid structure reported previously in the absence of ssRNA (11). Upon the addition of RNA^cognate^, there was a pronounced one-step increase in FRET efficiency from the unbound (∼0.4) to the ssRNA-bound (∼0.8) conformation of dPPR10. Initial binding of ssRNA was predominantly (80%) observed to be stable (i.e. no dynamic changes in FRET post initial binding) or, in rarer cases (20%), unstable (i.e. multiple FRET transitions are observed post initial binding) (Figure 2B). These dynamic events might be explained by the presence of a small subset of RNA^cognate^ heterodimers or misfolded dPPR10 proteins, which result in non-stable dPPR10-bound states. Notably, the proportion of dynamic molecules decrease over time (Supplementary Figure S5A); this likely since RNA^cogntate^ heterodimers can transiently associate/dissociate until a non-duplexed ssRNA species forms a stable interaction or the protein acquires the correct conformation over time. Importantly, dPPR10 adopted the high-FRET state (∼0.8) only when incubated in the presence of RNA^cognate^ and not when incubated in the presence of non-target RNA (i.e. RNA^anti-cognate^) (Figure 2D), demonstrating the specificity of dPPR10 for its target.

We further exploited the smFRET assay to investigate how mismatches at different regions of the ssRNA cognate sequence affect the affinity and specificity of dPPR10 binding. To do this, we introduced single and double nucleotide substitutions into RNA^cognate^ within the region of dPPR10 that is monitored by smFRET (denoted as RNA^U9A^, RNA^A10U^ and RNA^U9A, A10U^_,_ RNA^U8A^, where the number corresponds to the n^th^ nucleotide from the 5′ end) (Figure 2E and Supplementary Figure S5B). When dPPR10 was incubated in the presence of the single-nucleotide mismatched ssRNA, FRET trajectories dynamically transitioned between bound and unbound PPR conformations (Supplementary Figure S5C) and the FRET distributions were bimodal (Figure 2F). The double mismatched ssRNA (i.e. RNA^U9A, A10U^) was unable to induce any increase in FRET, with the individual traces and histograms appearing like that of dPPR10 in the absence of ssRNA and is consistent with SPR data (Figure 2G).

ssRNA constructs were also designed that contained mismatches outside the region of dPPR10 probed by smFRET at the 3′ end (denoted as RNA^U15A^, RNA^A14U^ and RNA^A14U, U15A^, RNA^U17A^) or 5′ end (denoted RNA^G2C^ and RNA^U3A^, RNA^U5A^) (Figure 2E and Supplementary Figure S5B). All ssRNA constructs were able to induce the high-FRET state characteristic of RNA-bound dPPR10 (Figure 2F and Supplementary Figure S5D); however, RNA species that contained mismatches at the 3′ end were more tolerated by dPPR10 compared to those that contain mismatches at the 5′ end or centrally within the binding site (Supplementary Figure S5E). The observed changes within the FRET distributions were consistent with SPR and ensemble-based FRET titrations (Figure 2G and Supplementary Figure S5F), whereby the amplitude of the SPR signal during the association phase scaled with the height of the high FRET peak in the corresponding single-molecule distributions (Figure 2D and F, and Supplementary Figure S5D), indicating that the conformational compaction of the N-terminal region of dPPR10 acts as an excellent global reporter for RNA binding. Dissociation of RNA^cognate^ measured by SPR was biphasic (Supplementary Figure S1C), revealing a minor fraction of unstable (fast dissociation) and a major fraction of stable complexes (slow dissociation), consistent with the subsets of stable (80%) and dynamic (20%) smFRET traces observed with RNA^cognate^. Notably, all ssRNA molecules that contained a mismatch formed unstable complexes that dissociated rapidly from dPPR10 when removed from solution, consistent with the frequent switching from the high to the low FRET state in the corresponding single-molecule traces. Since association and dissociation of RNA can occur concurrently during the association phase in SPR, the faster *k*_off_s for mismatched ssRNA appear to mostly explain the lower amplitude observed by SPR during association (note that *k*_on_s were not much perturbed, particularly for RNA^A14U^; Supplementary Figure S1C). These differences in dissociation kinetics were recapitulated using a combination of microfluidics and smFRET, whereby a decrease in FRET efficiency (indicative of ssRNA dissociation) upon removal of ssRNA from solution was only observed when dPPR10 had been pre-incubated and bound to a mismatched ssRNA (e.g. RNA^A14U^), but not RNA^cognate^ (Figure 2H). Taken together these results indicate that while nucleotides close to the 3′ do not have a critical role in initial binding of RNA to the expanded apo-dPPR10 structure, their relative contribution to binding becomes more prominent as the interaction(s) remodel during solenoid compaction.

To complement the SPR data, we performed kinetic analyses of the individual FRET trajectories by fitting the data to a HMM. The HMM allows discrete FRET states to be identified within the noise and the duration of each state (denoted as the residence time) to be quantified. For simplicity, FRET states were classified as either low-FRET (<0.5, corresponding to unbound PPR) or high-FRET (> 0.5, corresponding to ssRNA-bound PPR) and as such, transitions between FRET states can be filtered into two transition classes (high to low, denoted as T_high-low_, and low to high, denoted as T_low-high_). We note that T_high-low_ residence times can be reliably determined for ssRNA species containing mismatches only, since ssRNA containing a full cognate sequence exhibit dissociation times that are longer (∼1–2 h as determined by SPR) than can be observed by smFRET, due to photobleaching. Two of the three ssRNA constructs with middle mismatches (RNA^A10U^ and RNA^U9A, A10U^) had longer T_low-high_ residence times compared to the ssRNA constructs with mismatches in the region of dPPR10 not probed by FRET (i.e. RNA^U15A^, RNA^A14U^ and RNA^A14U, U15A^, since the PPR-motifs that ‘pair’ with these mismatches are more C-terminally located than the two cysteines labeled for FRET) (Supplementary Figure S1B and S5G). It should be noted that while residence times can be extracted from any observed binding or release event, for some RNA species (e.g. RNA^U9A, A10U^) the occurrence of such transitions are rare (<20). Since mismatches within the center of RNA^cognate^ substantially affect binding to dPPR10 (Supplementary Figure S5E), we hypothesized that binding is favoured when consecutive motif-nucleobase contacts are possible (since centrally positioned mismatches would lead to shorter nucleotide tracts at the 3′ and 5′ ends). To investigate this, we determined the longest consecutive tract of possible PPR-nucleotide cognate contacts for each mismatched ssRNA variant and calculated the proportion of dPPR10 molecules that are dynamic based on the HMM fits (i.e. those that exhibited both T_low-high_ and T_high-low_ transitions). Interestingly, regardless of the mismatch position (i.e. 5′ or 3′), shorter consecutive PPR-nucleotide tracts correlated with more dynamic dPPR10 molecules (Supplementary Figure S5H). Collectively, this data demonstrates that mismatches detrimentally affect the association of ssRNA to dPPR10 in a sequence position-dependent manner and that interrupting consecutive PPR-nucleotide contacts drastically decreases the stability of the PPR-RNA complex.

### Truncated RNA associates faster with dPPR10 while a minimum of 10 PPR-nucleobase contacts are required for conformational compaction

**Figure 3.**
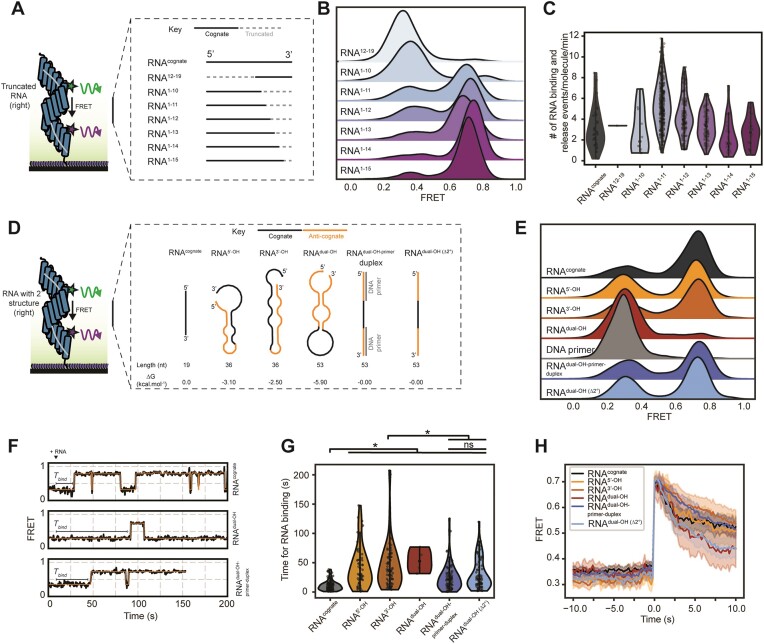
A minimum of 10 PPR-nucleobase contacts are required to induce conformational compaction of dPPR10 while RNA secondary structure regulates access to cognate sequence. (**A**) Schematic showing the various truncations to RNA^cognate^ that were tested using smFRET. (**B**) Ridgeline plot of the FRET distributions of dPPR10 incubated in the absence or presence of the indicated truncated ssRNA oligonucleotides (1 μM). Data is collated from at least 77 individual molecules. (**C**) Violin plots showing the distribution of ssRNA sampling events by individual dPPR10 molecules in the presence of the different truncated ssRNA oligonucleotides. Note that the rates determined for RNA^cognate^ represents the unstable fraction of complexes that represesent 20% of all traces. (**D**) Schematic of the various extended ssRNA oligonucleotides tested using smFRET and their predicted secondary structure propensities and structures. The position of RNA^cognate^ (*black lines*) and the presence of non-target RNA sequences (*lighter coloured lines*) are indicated for each ssRNA. Schematics not shown to scale. (**E**) Ridegeline plot of the FRET distributions of dPPR10 incubated in the absence or presence of the indicated ssRNA oligonucleotides (1 μM). Data is collated from at least 174 individual molecules. (**F**) Representative FRET trajectories of dPPR10 upon the injection of the indicated ssRNA (1 μM) after 10 s (indicated above the traces). The time taken until RNA binding (*T*_bind_) is shown for each trace and used to plot data in panel (G). (**G**) Violin plot of the time taken until ssRNA binding. The ssRNA was injected into the flow cell after 10 s and the time until the first transition was determined. A one-way ANOVA statistical analysis with Tukey’s multiple-comparisons *post-hoc* test was performed to determine statistically significant differences in *T*_bind_ between treatment groups. *P ≤ 0.05. ns or the absence of markers indicates no significant difference (*P* > 0.05). (**H**) The average FRET efficiency immediately prior to and after a T_low-high_ transition for dPPR10 in the presence of the indicated ssRNA oligos. T_low-high_ transitions were filtered for those that had residence times longer than 10 s.

Next, we sought to identify a minimum ssRNA sequence that would induce a conformational change in dPPR10. To do this, we utilized an ssRNA sequence that contained only 8 nt at the 3′ end of RNA^cognate^ (denoted RNA^12–19^) and a series of truncated ssRNA sequences containing the first 10–16 nt at the 5′ end of RNA^cognate^ (denoted RNA^1–10^ to RNA^1–16^) (Figure 3A). When dPPR10 was incubated in the presence of RNA^12–19^, the FRET histogram distributions were like that observed in the absence of ssRNA (Figure 3B), suggesting that dPPR10 is unable to stably interact with this truncated ssRNA target. Similarly, incubation of dPPR10 with the shortest 3′ truncation construct, RNA^1–10^, resulted in FRET distributions similar to dPPR10 in the absence of ssRNA. The addition of a single nucleotide (i.e. RNA^1–11^), however, resulted in individual dPPR10 molecules dynamically transitioning between low-FRET and high-FRET states (Supplementary Figure S6A) and the appearance of a bimodal FRET distribution (Figure 3B). This data was consistent with SPR results, whereby dPPR10 was not able to bind to RNA^12–19^ but could associate rapidly with RNA^1–11^ (albeit with a lower amplitude than RNA^cognate^); notably, RNA^1–11^ was observed to dissociate rapidly from dPPR10 upon removal of RNA from solution (Supplementary Figure S6B). As such, RNA^1-11^ represents a minimum ssRNA sequence required to induce conformational compaction of dPPR10, with the subsequent addition of nucleotides to this construct (i.e. RNA^1–12^ to RNA^1–16^) also able to induce stable transitions to the high-FRET state consistent with the ssRNA-bound dPPR10 state. To determine whether shorter RNA fragments can bind to and compact the corresponding segments of PPR proteins, RNA^1–12^ was further truncated from the 5′ end such that generated fragments spanned the region of dPPR10 probed by FRET (denoted RNA^1–12^–RNA^5–12^, encompassing PPR motifs 3–10). Removal of the first nucleotide that makes a PPR-motif contact (i.e. G2) abolishes conformational compaction, despite the fact that (i) this nucleotide lies outside the region probed by FRET and (ii) that there are 10 other nucleotides spanning the FRET-probed region that are still available for PPR-motif binding (Supplementary Figure S6E and F). Notably, this is the same number of available contacts possible for RNA^1–11^ (i.e. 10 nt, which does induce dPPR10 compaction), highlighting the importance of the first binding nucleotide. These data demonstrate two things; (i) having available nucleotides that span multiple PPR motifs is not always sufficient for compaction of that protein region and (ii) that nucleotides outside of the region to be probed by FRET can drastically affect compaction of that region. It remains to be tested whether other 11 nucleotide long sections RNA^cognate^ (in the middle or at the 3′ end) can similarly induce compaction, or whether the 5′ region is strictly required. Interestingly, combined incubation of RNA^1–11^ with RNA^12–19^, which together encompasses the entire RNA^cognate^ sequence, resulted in a similar proportion of high-FRET states compared to RNA^1–11^ alone and less than that observed for RNA^cognate^ (Supplementary Figure S6C). Since association of RNA^12–19^ to dPPR10 is exceedingly slow, the probability that both ssRNA species are bound to dPPR10 is low and as such the simultaneous presence of both sequences (i.e. as is the case for RNA^cognate^) is required for efficient binding. Notably, of the truncation constructs that could induce an increase in FRET, the shortest ssRNA sequence (i.e. RNA^1–11^) induced the highest frequency of switching between FRET states of dPPR10 and this switching frequency was observed to decrease with increasing length of ssRNA (Figure 3C). This is due to most truncated ssRNAs having significantly shorter T_low-high_ and T_high-low_ residence times (i.e. faster binding/dissociation rates) compared to the least truncated ssRNA (Supplementary Figure S6D), which indicates that shorter ssRNA sequences may have improved accessibility to dPPR10 (but lower stability) compared to longer sequences.

### Stable RNA secondary structures sequesters the target sequence and prevent dPPR10 binding

We next sought to investigate how the fusion of flanking non-target sequences to RNA^cognate^ affects the kinetics of dPPR10 binding. To do this, we implemented ssRNA constructs with extensions on either the 5′ or 3′ end (∼17 nt, denoted RNA^5’-OH^ or RNA^3’-OH^ respectively) or on both ends (RNA^dual-OH^). All of the extended ssRNA constructs have a higher predicted propensity to form local secondary structure (ΔG < −2.5 kcal/mol) relative to RNA^cognate^ (ΔG = 0.0 kcal/mol) (Figure 3D and Supplementary Figure S1B). Incubation of dPPR10 with either RNA^5’-OH^ or RNA^3’-OH^ resulted in the formation of the high-FRET state indicative of ssRNA binding (Figure 3E) and consistent with ensemble-based SPR data (Supplementary Figure S7A). Interestingly, only a small fraction of dPPR10 molecules occupied the high-FRET state when incubated in the presence of RNA^dual-OH^, indicating that the additional sequences flanking the cognate prevent (or slow down) its recognition and binding by dPPR10. The inability of dPPR10 to bind RNA^dual-OH^ could be either due to non-productive association with the additional sequence or exclusion from the cognate site, by, for example, RNA secondary structures. If either of these hypotheses are correct, we would expect preventing interaction between the extensions and the target site of dPPR10 to improve binding. Indeed, incubation of RNA^dual-OH^ duplexed with DNA primers annealed to the sequences flanking the cognate target site resulted in a stark increase in the fraction of molecules with high FRET efficiency to a level similar to that observed for RNA^cognate^ (Figure 3E and Supplementary Figure S7B). Incubation of the DNA primer alone did not result in an increase in FRET efficiency; indeed, native-PAGE analysis confirmed that the addition of DNA primer serves only to increase the binding preference of PPR to duplexed RNA^dual-OH^ species (Supplementary Figure S7C). To distinguish between association of dPPR10 with the extension sequences and association of the cognate target with the extension sequences as the explanation for the inhibition of binding, we prepared an ssRNA oligonucleotide of identical length to RNA^dual-OH^ (53 nt) but without predicted secondary structure (denoted as RNA^dual-OH (Δ2°)^). dPPR10 binds to this RNA as well as it binds to RNA^dual-OH^ duplexed with DNA primers (Figure 3E). This suggests that the key limitation to binding is not the length of the RNA species *per se*, nor association of the PPR with non-cognate sequence, but rather RNA secondary structure occluding the target site.

### Increased RNA transcript length and secondary structure propensity slows association to PPR

Using microfluidics, we determined the time taken until an increase in FRET was observed following injection of the RNA constructs into the flow cell containing dPPR10 (Figure 3F and G, additional traces in Supplementary Figure S7D). As expected, RNA^cognate^ was the fastest to induce an increase in FRET (mean binding time of 10.6 ± 0.8 s), with RNA^5’-OH^ and RNA^3’-OH^ taking four times longer (41.3 ± 4.9 and 43.4 ± 4.6 s, respectively) and RNA^dual-OH^ taking five times longer (53.0 ± 10.2 s) to cause an increase in FRET. However, when DNA primers were annealed to RNA^dual-OH^, dPPR10 was able to bind to the duplexed construct substantially faster with a mean binding time of 24.0 ± 2.2 s (i.e. two-fold that of RNA^cognate^, Figure 3G). dPPR10 was observed to bind to RNA^dual-OH (Δ2°)^ with a similar mean binding time (30.0 ± 3.0 s) compared to RNA^dual-OH^ duplexed to DNA primer, which suggests that the presence of flanking ssRNA (as opposed to PPR-inaccessible RNA:DNA duplexes) does not assist (but may delay) the dPPR10 protein in recognizing and binding to its cognate sequence within a longer RNA molecule. The slow binding kinetics of dPPR10 when incubated in the presence of all RNA extension constructs is supported by T_low-high_ residence times, which are similar to the corresponding binding times (Supplementary Figure S7E). These data extends on the previous observation that the length of an RNA transcript affects association rates (e.g. truncated ssRNA species associate to dPPR10 faster than RNA^cognate^), even in the absence of RNA secondary structure.

Interestingly, for all ssRNA constructs the T_low-high_ FRET transitions occurred abruptly from one frame to the next (Figure 3H), which suggests that the longer T_low-high_ residence times are not due to some rate-limiting conformational rearrangement of dPPR10 (which would result in a gradual increase in FRET efficiency). Intriguingly, smFRET experiments demonstrate that the presence of 100-fold excess of non-target RNA (i.e. RNA^anti-cognate^) did not affect the rate or amount of dPPR10 association to RNA^cognate^ (Supplementary Figure S7F and G), whereas non-target sequences that flank the cognate sequence (e.g. RNA^dual-OH (Δ2°)^) affect binding kinetics. These data indicate that the delay in dPPR10 conformational compression in the presence of longer sequences is not due to competition between the target and non-target sequences for dPPR10 recognition but is rather a topological issue, whereby dPPR10 likely has to associate with the target sequence in the correct orientation (i.e. 5′ to 3′ direction) without perturbation from non-target flanking sequences.

### dPPR10 does not ‘scan’ non-cognate sequences and binds directly to the cognate site

**Figure 4.**
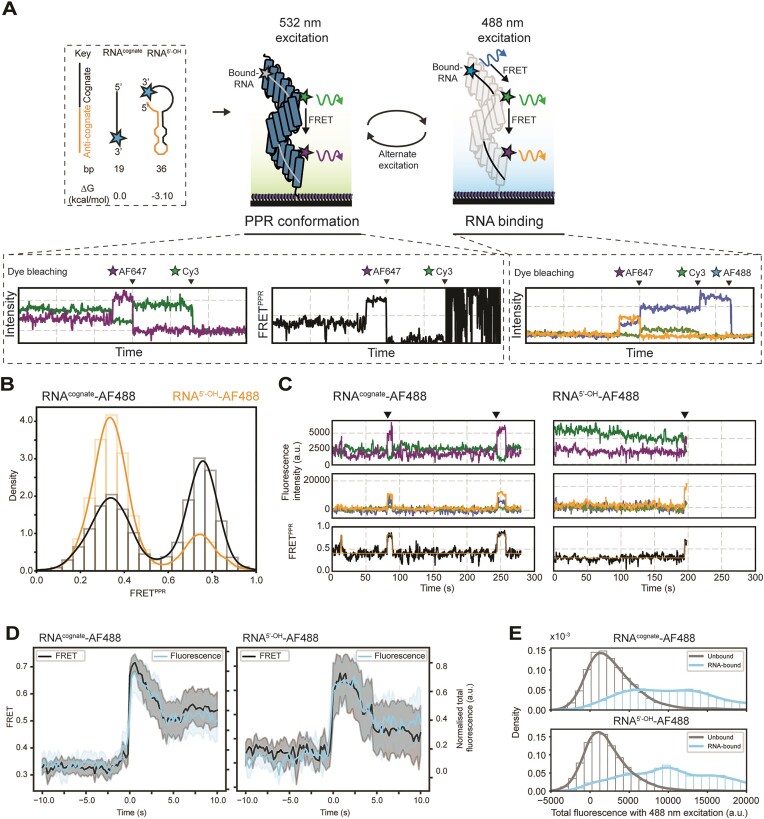
dPPR10 conformational compaction occurs only upon specific recognition and binding to the cognate sequence. (**A**) Schematic of the three-color smFRET experimental setup. Immobilized Cy3/AF647-labeled dPPR10 is incubated in the presence of AF488-RNA^cognate^ or AF488-RNA^5'-OH^ (20 nM) and alternatively excited with a 532 nm laser (to monitor changes in dPPR10 FRET) or 488 nm laser (to monitor association of labeled ssRNA to dPPR10) and the fluorescence emission of each dye is measured over time. For clarity, the point at which each of the dyes photobleaches during imaging is shown above each example trace. Example RNA molecules are not shown to scale. (**B**) FRET histogram of dPPR10 incubated in the presence of AF488-RNA^cognate^ or AF488-RNA^5'-OH^. Data is collated from at least 282 individual molecules. (**C**) Representative fluorescence intensity traces of dPPR10 and either AF488-FRET^cognate^ (*left*) or AF488-RNA^5'-OH^ (*right*) when excited with the 532 nm laser (*top*), the 488 nm laser (*middle*) and FRET^PPR^ trajectories upon excitation with the 532 nm laser (*bottom*). The arrows above the traces indicate events where an increase in FRET^PPR^ is correlated with the association of AF488-RNA. (**D**) Synchronized transition plots showing the PPR^FRET^ and normalized AF488-RNA^cognate^ (*left*) or AF488-RNA^5'-OH^ (*right*) total fluorescence immediately prior to and after a T_low-high_ transition (indicative of ssRNA binding). (**E**) The total fluorescence intensity of AF488, Cy3 and AF647 fluorophores upon 488 nm excitation depending on whether FRET^PPR^ is <0.5 (*unbound*) or > 0.5 (*RNA-bound*) in the presence of either AF488-RNA^cognate^ (*top*) or AF488-RNA^5'-OH^ (*bottom*). Note that data in E is inclusive of all proteins, not just those in which RNA binding events were observed [visualized in panel (**D**)].

It is interesting that we observed a substantial binding delay of dPPR10 to RNA sequences that contain secondary structure but do not detect any conformational rearrangement of the PPRs prior to compaction. To determine if we were missing any intermediate bound states where dPPR10 had not undergone conformational changes, we set out to perform three-color smFRET experiments with fluorescently labeled ssRNA to directly correlate ssRNA binding with dPPR10 conformational changes. To do so, immobilized dPPR10 was incubated with an AF488-labeled RNA^cognate^ (AF488-RNA^cognate^) and alternatively excited with a 532 nm laser (to monitor dPPR10 conformational changes using smFRET, denoted FRET^PPR^) and a 488 nm laser (to visualize the binding of labeled ssRNA) (Figure 4A). As expected, incubation of dPPR10 with AF488-RNA^cognate^ resulted in the formation of the high-FRET^PPR^ state (∼0.7) typical of ssRNA-bound dPPR10, indicating that the attachment of a fluorophore to RNA^cognate^ does not affect its ability to induce dPPR10 conformational changes (Figure 4B). Notably, the signal from the AF488-RNA molecule appeared in the same frame as the T_low-high_ FRET^PPR^ transition (Figure 4C), indicating that ssRNA binding immediately led to dPPR10 compaction. Further, excitation with the 488 nm laser led to a concurrent increase in the total fluorescence of all three dyes associated with the dPPR10-ssRNA system (i.e. AF488, Cy3 and AF647), indicating that AF488-RNA^cognate^ is participating in FRET with the fluorophores conjugated to dPPR10 (Figure 4C and D, and Supplementary Figure S8). Likewise, transitions to low-FRET^PPR^ states (i.e. <0.5) were coincident with a loss of fluorescence from all dyes upon excitation at 488 nm (Figure 4C). Interestingly, similar results were observed for AF488-RNA^5’-OH^, where although AF488-RNA^5’-OH^ did not bind to dPPR10 as efficiently as the cognate (Figure 4B), the increases in FRET^PPR^ observed were directly correlated with an increase in dye fluorescence following excitation at 488 nm (Figure 4C and D). Notably, in the presence of either labeled ssRNA constructs, when dPPR10 exists within the low-FRET^PPR^ state (i.e. apo-dPPR10) the total fluorescence of all of the dyes was close to zero upon excitation at 488 nm; conversely, when dPPR10 resided in the high-FRET^PPR^ state (i.e. ssRNA-bound dPPR10) the total fluorescence of all the dyes was substantially higher (Figure 4E). Collectively, these data indicate that the association and dissociation of ssRNA to dPPR10 is directly correlated with its observed transitions to a conformationally compact or relaxed state, respectively. Furthermore, since a non-specific ssRNA-dPPR10 intermediate complex could not be observed, this data demonstrates that dPPR10 binds directly to the cognate sequence and has limited or no capacity to ‘scan’ ssRNA transcripts. Such a binding model provides a simple explanation as to why sequestration of cognate sequences within secondary structure elements can effectively prevent dPPR10 binding.

## Discussion

We present a crystal structure of a P-class designer PPR protein bound to a target RNA molecule and used this system to develop and characterize a robust PPR-based FRET sensor for RNA binding. We then interrogated the effects of RNA modifications (e.g. mismatches and secondary structure) on dPPR10 binding using single-molecule fluorescence approaches. We demonstrate that dPPR10 becomes conformationally compressed when bound to RNA, consistent with previous reports of other PPR proteins (12,14), and that this compaction is supported by contact of the RNA phosphate with Lys13 within each motif. Furthermore, smFRET and SPR data revealed that mismatches within the center of the binding site, but not the 3′ end, of the cognate sequence severely impairs association to dPPR10 and that a minimum of 10 PPR-nucleobase contacts are required to induce binding to dPPR10. Notably, two- and three-color smFRET highlights that RNA secondary structure reduces the accessibility of binding sites to dPPR10.

It has been proposed that PPR10 regulates the translation of *atpH* by preventing the formation of secondary structure directly upstream of the ribosome binding site (7), and prevents nuclease attack (5), with upstream binding of start codons also observed for other PPR proteins (42,43). Paradoxically, however, the binding of PPR to RNA sequences containing secondary structure elements is impaired *in vitro* (44), which begs the question as to how PPR deals with such challenges. The data reported in this work supports a model whereby the presence of RNA secondary structure slows or prevents dPPR10 binding by sequestering the cognate sequence such that it cannot be readily accessed by dPPR10, which itself has no ability to bind to these structures. Our data, as well as others, indicate binding to secondary structure elements does not occur since PPR motifs make direct contact with the face of the RNA nucleobase (12,45). Indeed, even when portions of the cognate sequences are accessible within modelled RNA structures (46), binding to these sequences remained impaired compared to RNA^cognate^; which suggests that secondary structures introduce entropic constraints that may interfere with key PPR-ssRNA contacts (i.e. Lys13, amino acids at position 5 and 35) that enable initial recognition and binding. This is consistent with our structural data, whereby ssRNA is bound in an essentially linear (albeit helical) manner as it is threaded through the PPR cavity; as such, extreme curvature of the cognate sequence imposed by secondary structure likely prevents ssRNA rearrangments that are optimal for dPPR10 binding.

As such, the observed delay in dPPR10 binding to ssRNA sequences containing predicted secondary structure is likely due to the kinetics of ssRNA secondary structure formation, disappearance or interconversion between different possible secondary structures. The finding that even relatively weak ssRNA secondary structures (ΔG = ∼−2.5 kcal/mol) have a substantial effect on the ability of dPPR10 to bind to target sequences is somewhat surprising considering that certain PPR proteins, including PPR10, are known to bind at sites *in vivo* that can form hairpin structures (47,48). Notably, PPR10 and PPR53 binds to sites *in vivo* that have similar or higher secondary structure stability (ΔG = −2.8 or −8.0 kcal/mol, respectively) compared to the ssRNA variants used in this work (7,47). Indeed, the stability of these secondary structures are likely to be substantially higher *in vivo* compared to *in vitro* due to macromolecular crowding, which is known to promote folded and compact ssRNA structures (49,50). The reduced ability of dPPR10 to bind to sites containing secondary structure appears to be in conflict with its proposed mechanism of translation upregulation; it is likely that PPR proteins performs this role in cooperation with other proteins *in vivo*, such as RNA helicases, chaperones or sRNAs (51), that may temporarily resolve secondary structure elements to facilitate stable PPR binding; investigating the effect of crowding on PPR binding (either *in vitro* or *in vivo*) would be an exciting area of future research.

Despite the well-established role of PPR proteins in RNA regulation, the question as to how they find their target sequences in the complex cellular milleu within biologically relevant timescales remains an under-explored area of study. Two models are possible; either a PPR protein binds to sequences upstream or downstream of the target sequence and ‘scans’ the ssRNA transcript until the target is encountered [2D search, fast association (known as facilitated diffusion)] or it opportunistically binds to exposed target sequences (3D search, slow association). The data presented in this study supports the latter model, since we do not observe any transient association of dPPR10 to longer ssRNA sequences prior to conformational compaction as observed using three-color smFRET. Furthermore, RNA^anti-cognate^ did not show any capacity to bind dPPR10, even transiently as determined by SPR and smFRET, which again suggests that dPPR10 cannot associate with non-target sequences. For proteins restricted to 3D diffusion-dependent searching, high protein concentrations are favoured since the probability of direct interaction with the target sequence is increased (52); conversely, excess non-target sequences reduce the probability of direct protein-target collisions and decreases association rates. Interestingly, direct binding to sequence-specific sequences (facilitated primarily by 3D diffusion) by nucleic-acid-binding proteins has been experimentally observed even in the presence of massive excess of non-target flanking sequences (53). It was proposed that direct binding is enhanced by unusual nucleic-acid structural features close to the target sequence, which improves protein accessibility or specific-binding propensity (53). It is tempting to speculate that local RNA structures also act to improve the probability of specific PPR binding; for example, RNA secondary structures disproportionally decrease the number of single-stranded, non-specific target sequences (owing to their increased abundance), which biases accessible binding sites towards target sequences and reduces the effective ‘search space’ for productive PPR binding.

**Figure 5.**
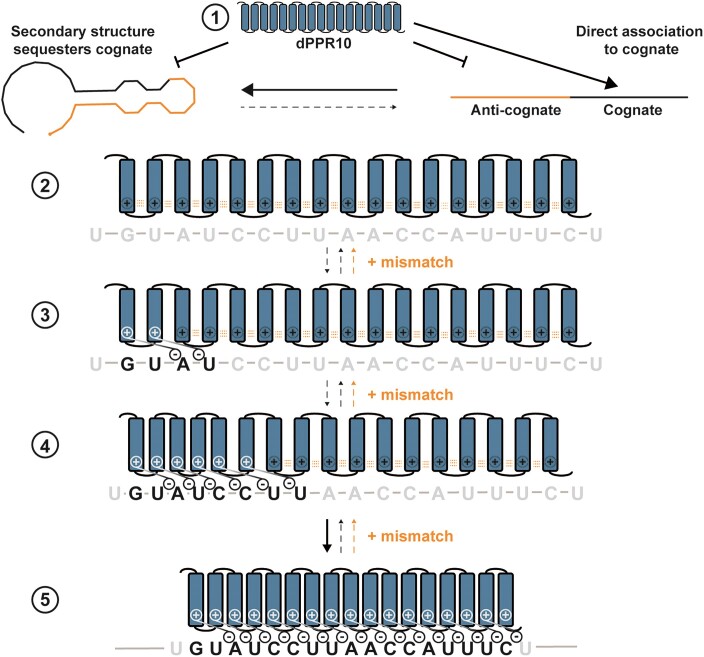
Binding mechanism of dPPR10 to ssRNA. (1) The presence of stable ssRNA secondary structure sequesters the cognate sequence, which prevents the association of dPPR10. If the stability of ssRNA secondary structure is sufficiently low, it can stochastically sample folded and unfolded configurations to which the dPPR10 will opportunistically associate with the transiently exposed cognate sequence (but not non-target ssRNA sequence). (2) apo-dPPR10 exists in a conformationally expanded state, driven by coulombic repulsion (*dotted lighter coloured lines*) between Lys13 residues in adjacent PPR motifs. (3) Initial PPR-nucleobase recognition is made (*black*), which stabilizes an electrostatic bridge between Lys13 in PPR module p and the phosphate moiety in nucleotide *b + 2*. For simplicity, binding to the 5′/N-terminal end is shown in this graphic; however, binding can possibly be initiated at the 3′/C-terminal end also. (4) Charge neutralization caused bt the Lys13–phosphate interaction reduces the coloumbolic repulsion that is common in the apo-dPPR10 conformation, resulting in local compression of PPR motifs that enables additional PPR-nucleobase and Lys13–phosphate interactions to be established. (5) At this stage, the charge neutralization effect becomes strongly favoured (due to the established multivalent interactions), which then propogates throughout the dPPR10 structure for stable binding. The presence of mismatches (or truncations) can significantly increase the reverse reaction.

We propose the following mechanism of dPPR10 recognition and binding to ssRNA (Figure 5). First, secondary structures sequester the cognate nucleobases from PPR motifs, preventing binding (Figure 3D–H and 4B and C). Transient exposure of the cognate sequence, the rate and propensity of which is primarily dictated by the inherent kinetics of RNA secondary structure and formation *in vitro* and possibly by other cofactors *in vivo*, allows cognate recogntion by PPR. Second, the initial contact of dPPR10 to RNA is via direct association of 2–3 PPR motifs with the cognate nucleobases and does not require ‘scanning’ of non-cognate sequences to do so. This is evidenced by several key findings, including (i) initial recognition of ssRNA by dPPR10 cannot be via non-specific lysine interactions with the RNA backbone, since the presence of excess non-target ssRNA species does not affect the rate of or binding to cognate (Supplementary Figure S7F and G); (ii) dPPR10 does not associate at all to non-cognate sequences (Figure 2D, Figure 4B and C, and Supplementary Figure S7F and G); and (iii) dPPR10 binds to RNA^dual-OH^ duplexed to DNA primer at similar rates as RNA^dual-OH (Δ2°)^, which indicates that there is no improved capacity of dPPR10 to search for the cognate if it is flanked by ssRNA vs duplexed nucleic acid (Figure 3D–H). Third, establishing a minimum of two PPR-nucleobase coordinates the interaction between Lys13 of the first bound PPR motif (*p*) with the phosphate backbone of a downstream nucleobase (*b + 2*), which results in charge neutralization of the lysine that annuls its repulsive force with Lys13 in the adjacent PPR module. Consequently, the adjacent module can partially contract to cooperatively establish contacts with the next nucleotide within the ssRNA sequence; this cycle of charge neutralization and motif compaction then propagates throughout the entire protein. Such a process is likely to be more efficient when initiated at the 5′ end (since charge neutralization of a downstream nucleotide would help coordinate contacts between the nucleobase and its corresponding PPR motif); however, based on our data it cannot be concluded if binding exclusively occurs in a 5′ to 3′ direction or vice versa. As such, it is possible that propogation can occur in either direction and might explain why centrally positioned mismatches often result in rapid association/dissociation events. Mutagenesis of Lys13 completely abolishes binding of PPR to ssRNA (12), which supports our proposed model that a combination of specific amino-acid/nucleobase and Lys13–phosphate interactions are essential in overcoming the energy barrier for conformational rearrangements and stable ssRNA binding to dPPR10. Disruption of PPR motif and nucleobase interactions (e.g. by double mismatches) can disrupt the propogation of motif compaction upon binding (Figure 2F and G) or promote erronous motif expansion during dissociation (Figure 2G and H), which substantially reduces the *K*_D_. Notably, combined incubation of truncation variants does not restore the conformational compaction of dPPR10 to levels observed for RNA^cognate^, indicating that stable binding occurs only when a single continuous RNA molecule is present to coordinate the propagation of conformational changes throughout the entire protein.

RNA- or DNA-binding proteins typically bind to their target sequences faster than random, 3D diffusion would predict (54,55); this has been proposed to occur via facilitated difussion, whereby slow, non-specific association of protein to nucleic acid (3D diffusion dependent) is followed by faster 2D scanning (via hopping or sliding) until the cognate sequence is encountered (52). Since dPPR10 does not associate appreciably with non-cognate RNA, 2D scanning is restricted and is instead more dependent on 3D diffusion. The kinetic constraints imposed by random 3D diffusion may explain the longstanding maxim and associated conundrum of why PPR proteins are abundant in organelles (56–58), but almost unknown outside them (i.e. in the cytosol and nucleus). Other functionally analogous but non-homologous RNA binding protein families [e.g. mTERF proteins (59), HPT proteins (60), OPR proteins (61)] show a similar restriction to organelle compartments, perhaps for the same reason. Collectively, these findings have significant implications for the design of synthetic PPR proteins. Careful consideration must be given to the local binding environment (e.g. primary and secondary structure of RNA target) within the global transcriptome. As reported by others (18), dPPR10 binding appears to be relatively tolerant to mismatches toward the 3′ end compared to those within the center of the PPR binding site or 5′ end; however, single mismatches dramatically increase dissociation rates (irrespective of sequence position). While seemingly a negative feature, transient interactions of a PPR with an RNA transcript may be beneficial in some applications (i.e. to track localization of transcripts *in vivo* without perturbing function or transcript lifetime) but could be detrimental to others if stable binding is preferred (e.g. if attempting to modulate gene expression). Furthermore, PPR binding is surprisingly affected (or even completely abolished) by even weak secondary structure elements (ΔG = ∼−2.5 kcal/mol); as such, the design of synthetic PPRs should be targeted to RNA sequences within an RNA transcript that contains low predicted secondary structure. Finally, our data indicates that dPPR10 has a limited capacity to ‘search’ ssRNA transcripts for its target sequence. As such, this may have cautionary implications for the design of and use of synthetic PPRs in nuclear and cytoplasmic settings, restricting the use of dPPRs within organelle compartments, unless other approaches can be developed to increase the scanning speed of PPRs for their targets (e.g. fusing an RNA helicase to a PPR module).

## Supplementary Material

gkae984_Supplemental_File

## Data Availability

All data needed to evaluate the conclusions in the paper are present in the paper and/or the Supplementary Materials. All unique reagents generated in this study are available from the lead contact without restriction. Single-molecule and other raw data materials needed to evaluate the conclusions in the paper has been deposited at Zenodo at the following DOI:10.5281/zenodo.11323228.
