# Supplementary material for: Single-molecule visualization of sequence-specific RNA binding by a designer PPR protein
Source: Nucleic Acids Res. 2024 Nov 12;52(22):14154–70. doi: 10.1093/nar/gkae984 (PMC11662938; doi:10.1093/nar/gkae984)
Supplement: gkae984_Supplemental_File [file gkae984_supplemental_file.pdf]

Supplementary Materials

Supplementary Figures

A

	5' → 3'
$RNA^{cognate}$	UGUAUCCUUAACCAUUUCU
$RNA^{anti\text{-}cognate}$	UCACAGGAACCGGCAAAGU
RNA^{U8A}	UCUAUCCUUAACCAUUUCU
RNA^{U3A}	UGAAUCCUUAACCAUUUCU
RNA^{U5A}	UGUAACCUUAACCAUUUCU
RNA^{U8A}	UGUAUCCAUAACCAUUUCU
RNA^{U9A}	UGUAUCCUAAACCAUUUCU
RNA^{A10U}	UGUAUCCUUUACCAUUUCU
$RNA^{U9A,A10U}$	UGUAUCCUAUACCAUUUCU
RNA^{U15A}	UGUAUCCUUAACCAAUUCU
RNA^{A14U}	UGUAUCCUUAACCUUUUCU
$RNA^{A14U, U15A}$	UGUAUCCUUAACCUAUUCU
RNA^{U17A}	UGUAUCCUUAACCAUUACU
RNA^{1-10}	UGUAUCCUUA
RNA^{1-11}	UGUAUCCUUAA
RNA^{1-12}	UGUAUCCUUAAC
RNA^{1-13}	UGUAUCCUUAACC
RNA^{1-14}	UGUAUCCUUAACCA
RNA^{1-15}	UGUAUCCUUAACCAU
RNA^{12-19}	CCAUUUCU
RNA^{2-12}	GUAUCCUUAAC
RNA^{3-12}	UAUCCUUAAC
RNA^{4-12}	AUCCUUAAC
RNA^{5-12}	UCCUUAAC
$RNA^{5'\text{-}OH}$	CACAGGAACCGGCAAAGUGUAUCCUUAACCAUUUCU
$RNA^{3'\text{-}OH}$	UGUAUCCUUAACCAUUUCUCACAGGAACCGGCAAAG
$RNA^{dual\text{-}OH}$	CACAGGAACCGGCAAAGUGUAUCCUUAACCAUUUCUCACAGGAACCGGCAAAG
$RNA^{dual\text{-}OH\ (\Delta 2^{o})}$	CAUUCCAACCCCCAAAGUGUAUCCUUAACCAUUUCUCUCACCAACCCCCAAAG

B

	$T_{low\text{-}high}$ (s)	Relative binding	ΔG (kcal.mol^{-1})
$RNA^{cognate}$	6.1 ± 0.5	+++	0.00
$RNA^{anti\text{-}cognate}$	N/A	-	0.00
RNA^{U8A}	6.2 ± 0.3	+	0.00
RNA^{A9U}	10.7 ± 0.6	+	0.00
$RNA^{U8A,A9U}$	19.1 ± 5.9	-	0.00
RNA^{U15A}	5.5 ± 0.4	++	0.00
RNA^{A14U}	4.9 ± 0.2	++	0.00
$RNA^{A14U, U15A}$	4.9 ± 0.3	++	0.00
RNA^{1-10}	6.9 ± 0.9	-	0.00
RNA^{1-11}	3.4 ± 0.1	+	0.00
RNA^{1-12}	3.6 ± 0.1	+	0.00
RNA^{1-13}	3.5 ± 0.1	++	0.00
RNA^{1-14}	3.9 ± 0.6	++	0.00
RNA^{1-15}	7.0 ± 0.7	++	0.00
RNA^{12-19}	15.7 ± 7.1	-	0.00
$RNA^{5'\text{-}OH}$	40.1 ± 5.1	+	**-3.10**
$RNA^{3'\text{-}OH}$	29.5 ± 2.4	+	**-2.50**
$RNA^{dual\text{-}OH}$	42.1 ± 1.5	-	**-5.90**
$RNA^{dual\text{-}OH\ (\Delta 2^{o})}$	26.3 ± 2.0	++	0.00

C

	k_{on} (M^{-1}.s^{-1})	$\tau 1_{off}$ (s)	$\tau 2_{off}$ (s)	k_{off} (s^{-1})	K_D (nM)
$RNA^{cognate}$	1.22*10^{5}	86.1±0.30	7990±10.00	1.43*10^{-5}	1
$RNA^{anti\text{-}cognate}$	N/A	N/A	N/A	N/A	N/A
RNA^{U8A}	2.19*10^{4}	85.5±0.30	20.7±0.04	2.66*10^{-2}	1214
RNA^{A9U}	1.50*10^{4}	56.0±0.20	182.7±0.04	8.42*10^{-3}	561
$RNA^{U8A,A9U}$	N/A	N/A	N/A	N/A	N/A
RNA^{U15A}	2.70*10^{4}	73.4±0.20	250.3±0.30	5.21*10^{-3}	192
RNA^{A14U}	1.17*10^{5}	65.0±0.20	243.5±0.20	5.11*10^{-3}	43
$RNA^{A14U, U15A}$	7.60*10^{4}	66.6±0.20	204.2±0.20	6.30*10^{-3}	82
RNA^{1-11}	3.10*10^{4}	66.7±0.40	11.84±0.03	4.78*10^{-2}	1542
RNA^{12-19}	N/A	N/A	N/A	N/A	N/A
$RNA^{5'\text{-}OH}$	1.90*10^{4}	160.7±0.40	4670±10.0	2.67*10^{-4}	14
$RNA^{3'\text{-}OH}$	2.80*10^{4}	108.3±0.20	5071±9.00	2.61*10^{-4}	9

Figure S1: Schematic and sequence of each ssRNA oligonucleotide used in this work with summarised kinetic information. (A) The identity and sequence of each ssRNA oligonucleotide is shown, with modifications to $RNA^{cognate}$ (*orange*) and position of complementarity with DNA primers (*underlined*) denoted. **(B)** The $T_{low\text{-}high}$ residence times (indicative of ssRNA binding) determined for each ssRNA variant as determined by smFRET, reported as the mean ± SEM. The relative binding efficiency (fraction of molecules in the high FRET state in the FRET efficiency histograms) and propensity of each ssRNA to form secondary structure is also shown. **(C)** Association and dissociation rates of various ssRNAs, with calculated K_D, as determined using SPR. Dissociation curves were fit with a two-phase exponential decay function.

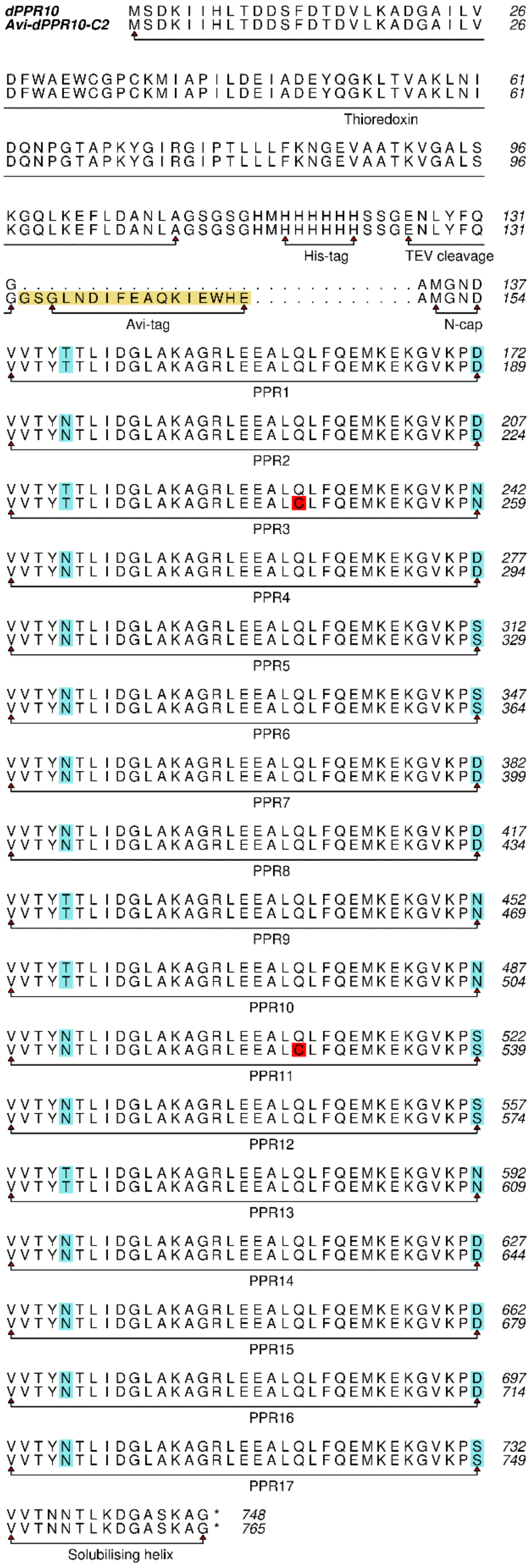

Figure S2: Details of protein sequences. Notable features are labelled or coloured (red indicates positions of cysteines introduced for labelling; cyan indicates RNA-specificity changes; yellow indicates the Avi-tag).

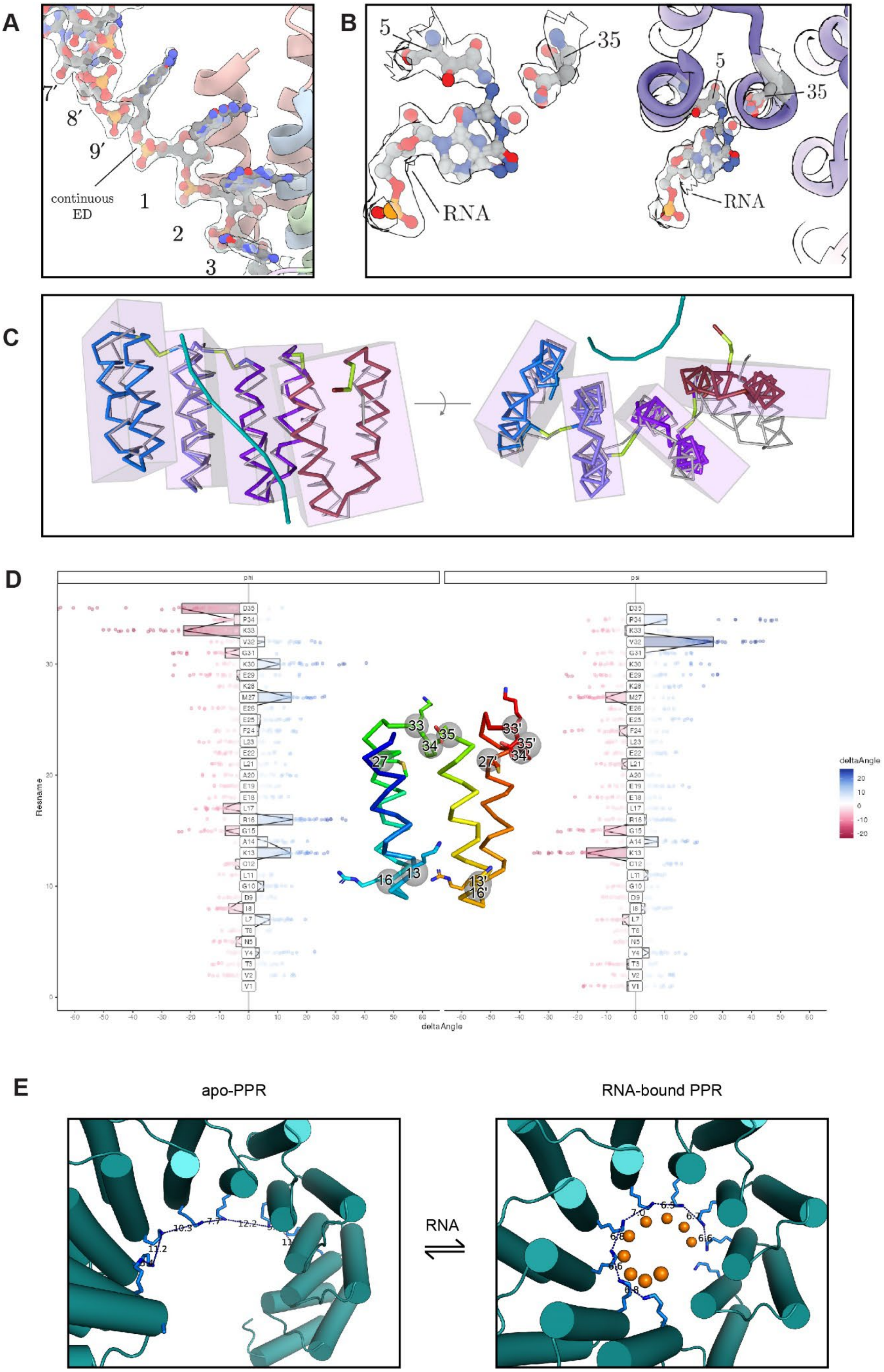
A
7′
8′
9′
continuous ED
1
2
3
B
5
35
RNA
5
35
RNA
C
D
phi
psi
Resname
deltaAngle
deltaAngle
20
10
0
-10
-20
E
apo-PPR
RNA-bound PPR
RNA

Figure S3: Structural features of PPR-protein:RNA binding. **(A)** Helical disorder in the structure of dPPR10:RNA complex results in fully connected eletron density between unit cells, and **(B)** microheterogeneity at the specific positions where nucleobases interact with residues 5 and 35. The structure otherwise has excellent geometry and electron density, representing an indeterminately long protein binding to an equally long RNA. **(C)** PPR repeats act as relatively rigid units (enclosed in transparent boxes) with a hinge region (green ribbon) between repeats, as analysed with DYNDOM. **(D)** Backbone geometry analysis (difference ϕ and ψ values at each position of a PPR repeat in the presence or absence of RNA, averaged across all repeats) reveals differences in protein conformation at only a small number of positions between apo- and ssRNA-bound structures (highlighted in inset), mainly corresponding to the hinge region. **(E)** Structural rearrangment in the presence of polyanionic RNA allow conserved cationic lysine sidechains to move closer together, a feature which may relate protein:RNA affinity to conformational change on binding.

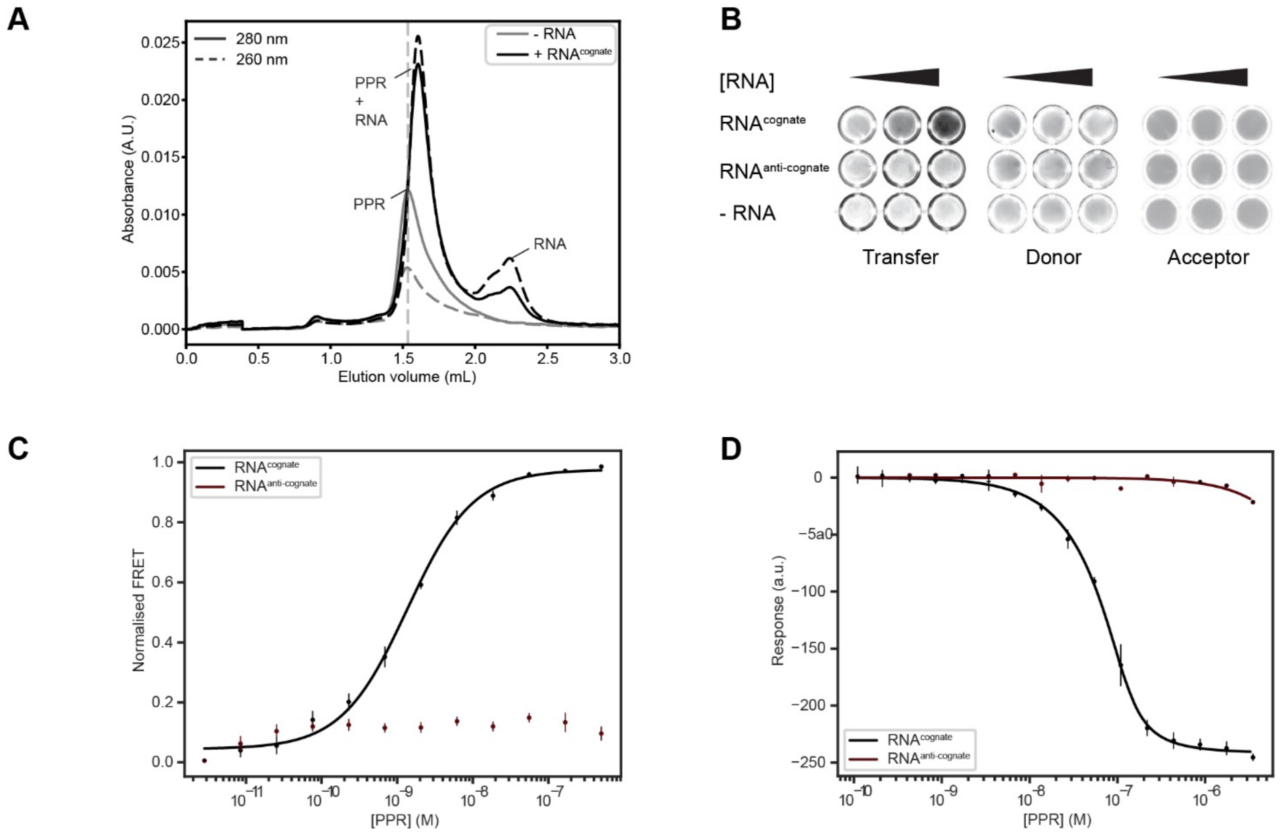

Figure S4: The conformational compaction of dPPR10 upon binding to ssRNA can be monitored in solution. (A) dPPR10 (15 µM) in the absence or presence of $RNA^{cognate}$ (18 µM) was analysed using analytical size exclusion chromatography (Superdex 200) revealing shift to higher elution volumes and the relative decrease in hydrodynamic radius upon the addition of ssRNA. **(B)** Representative wells of a fluorescently imaged 96-well microtitre plate, showing the increase in fluorescence of the acceptor dye when exciting the donor in the presence of increasing concentrations of $RNA^{cognate}$ (0.01 – 1000 nM). **(C)** Calculated FRET efficiency of labelled dPPR10 protein in the presence of either $RNA^{cognate}$ or $RNA^{anti\text{-}cognate}$, calculated from the bulk-FRET plate assay in (C). The K_D for $RNA^{cognate}$ was calculated to be 1.2 ± 0.2 nM (average of 3 technical replicates). FRET was normalised to the maximum measured value for dPPR10 in the presence of $RNA^{cognate}$. **(D)** Microscale thermophoresis of the fluorescently labelled dPPR10 protein, with $RNA^{cognate}$ and $RNA^{anti\text{-}cognate}$, showing a calculated binding affinity of 9.7 ± 1.8 nM (average of 3 technical replicates).

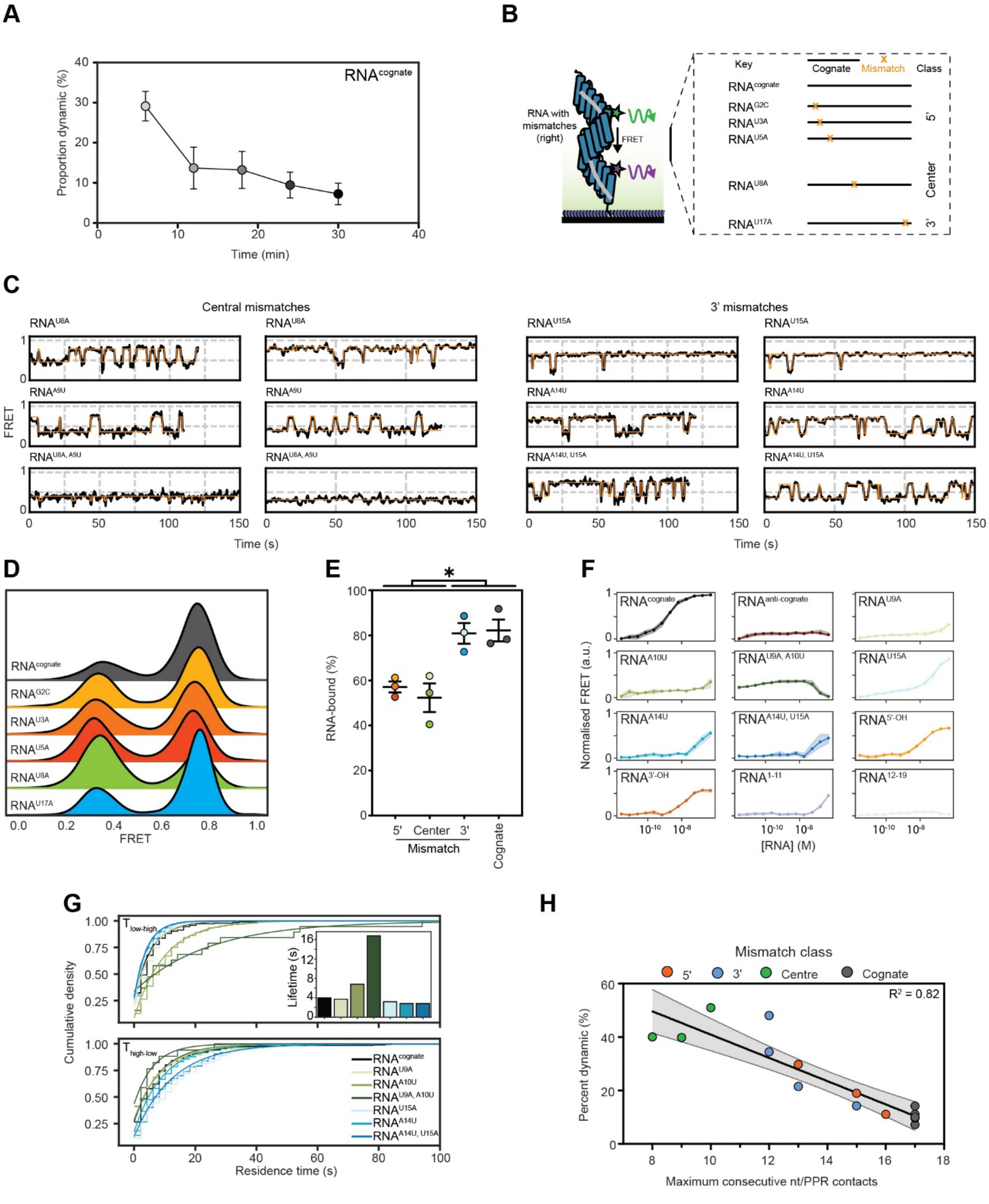

Figure S5: dPPR10 tolerates mismatches toward the 3' end of ssRNA but not when mismatches are located at the 5' end or centrally in the PPR binding site. (A) Proportion of dPPR10 molecules that are dynamic (i.e., those that exhibited both $T_{low\text{-}high}$ and $T_{high\text{-}low}$ transitions) when incubated in the presence of $RNA_{cognate}$ (1 µM) over time. Data is the mean ± SEM from 3 independent repeats. **(B)** Schematic showing the position of different mismatches introduced into $RNA^{cognate}$ for subsequent smFRET experiments. The key denotes the sequences that are identical (*black*) or different (*orange*) to $RNA^{cognate}$. **(C)** Representative FRET trajectories from individual dPPR10 molecules in the presence of the indicated ssRNA oligo containing mismatches within the dPPR10 region probed by FRET (1 µM, *left*) or at the 3' end (1 µM, *right*). Orange lines represent the fit from the HMM. **(D)** FRET histogram of dPPR10 incubated in the absence or presence of the indicated ssRNA oligonucleotides (1 µM) from B. Data is collated from at least 262 individual molecules. **(E)** The proportion of time

that dPPR10 is bound by ssRNA (i.e., > 0.5 FRET) containing mismatches at different positions. Only ssRNAs containing a single mismatch were included in this analysis and were grouped evenly according to their sequence position, which are defined as 5’ (nucleotides 1-6), center (nucleotides 7-13) or 3’ mismatches (nucleotides 14-19). A one-way ANOVA with Tukey’s post-hoc test was performed, with * indicating statistical significance ($p < 0.05$). **(F)** Ensemble-based FRET titration plots of dPPR10 in the presence of various ssRNA constructs used throughout this study. **(G)** Cumulative histogram of the residence time of different transition classes. Data is fit with a one-phase association curve. For $T_{low\text{-}high}$ residence times, the lifetimes calculated from the fit are shown. **(H)** The proportion of dPPR10 molecules that were observed to be dyna as a function of available consecutive nucleotide-PPR contacts. This was determined for each full length ssRNA as the longest consecutive number of cognate-like PPR-nucleotide contacts until a mismatch is encountered (if present). Each datapoint represents a single ssRNA construct and is calculated from data containing > 150 molecules and is fit with a linear regression.

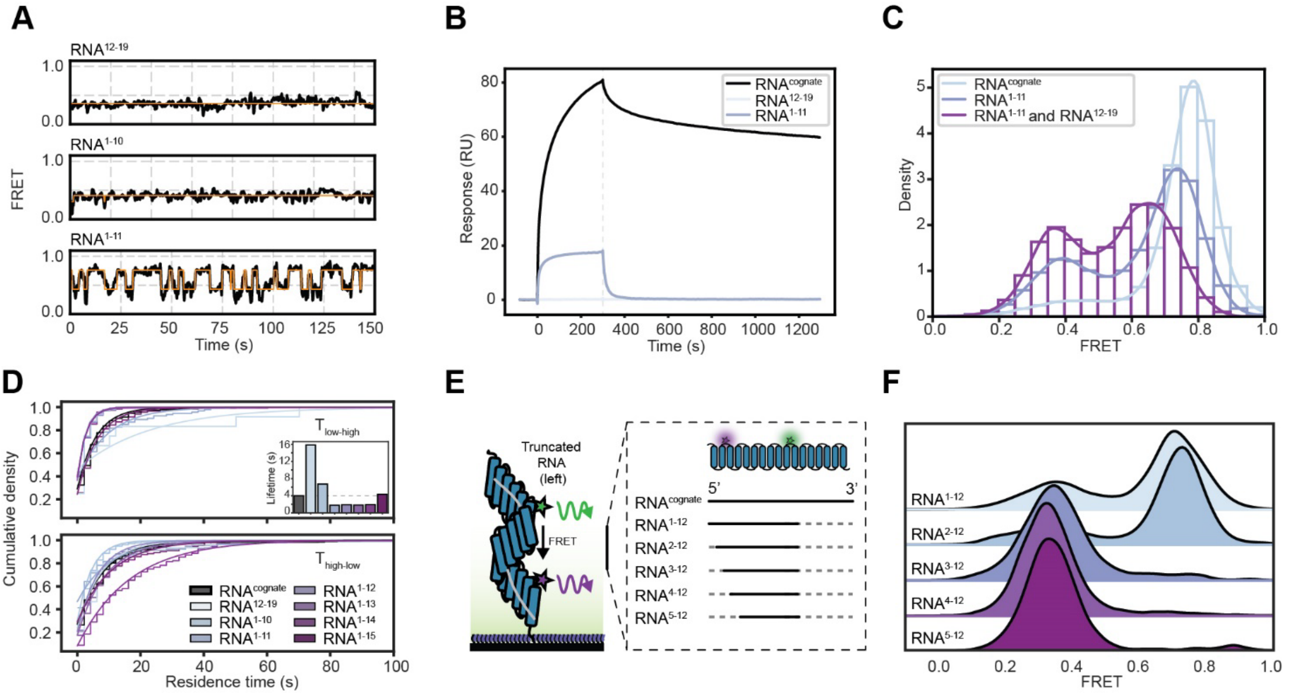

Figure S6: A minimum of 10 PPR-nucleobase contacts are required to induce conformational compaction of dPPR10. **(A)** Representative FRET trajectories from individual dPPR10 molecules in the presence of the indicated ssRNA oligo truncations (1 µM). Orange lines represent the fit from the Hidden Markov Model (HMM). **(B)** SPR association and dissociation curves of $\text{RNA}^{\text{cognate}}$, $\text{RNA}^{1\text{-}11}$ and $\text{RNA}^{12\text{-}19}$ binding to dPPR10. **(C)** FRET histogram of dPPR10 incubated in the presence of $\text{RNA}^{\text{cognate}}$ (1 µM), $\text{RNA}^{1\text{-}11}$ (1 µM), or a combination of $\text{RNA}^{1\text{-}11}$ and $\text{RNA}^{12\text{-}19}$ (1 µM each). **(D)** Cumulative histogram of the residence time of different transition classes. Data is fit with a one-phase association curve. For $T_{\text{low-high}}$ residence times, the lifetimes calculated from the fit are shown, with the dotted line indicating the value for $\text{RNA}^{\text{cognate}}$ (*inset*). **(E)** Schematic showing the various truncations to $\text{RNA}^{1\text{-}12}$ that were tested using smFRET. The position of the fluorophores used for smFRET are shown on dPPR10 and are aligned with their corresponding position on the ssRNA truncations. **(F)** Ridegeline plot of the FRET distributions of dPPR10 incubated in the absence or presence of the indicated ssRNA oligonucleotides (1 µM). Data is collated from at least 16 individual molecules.

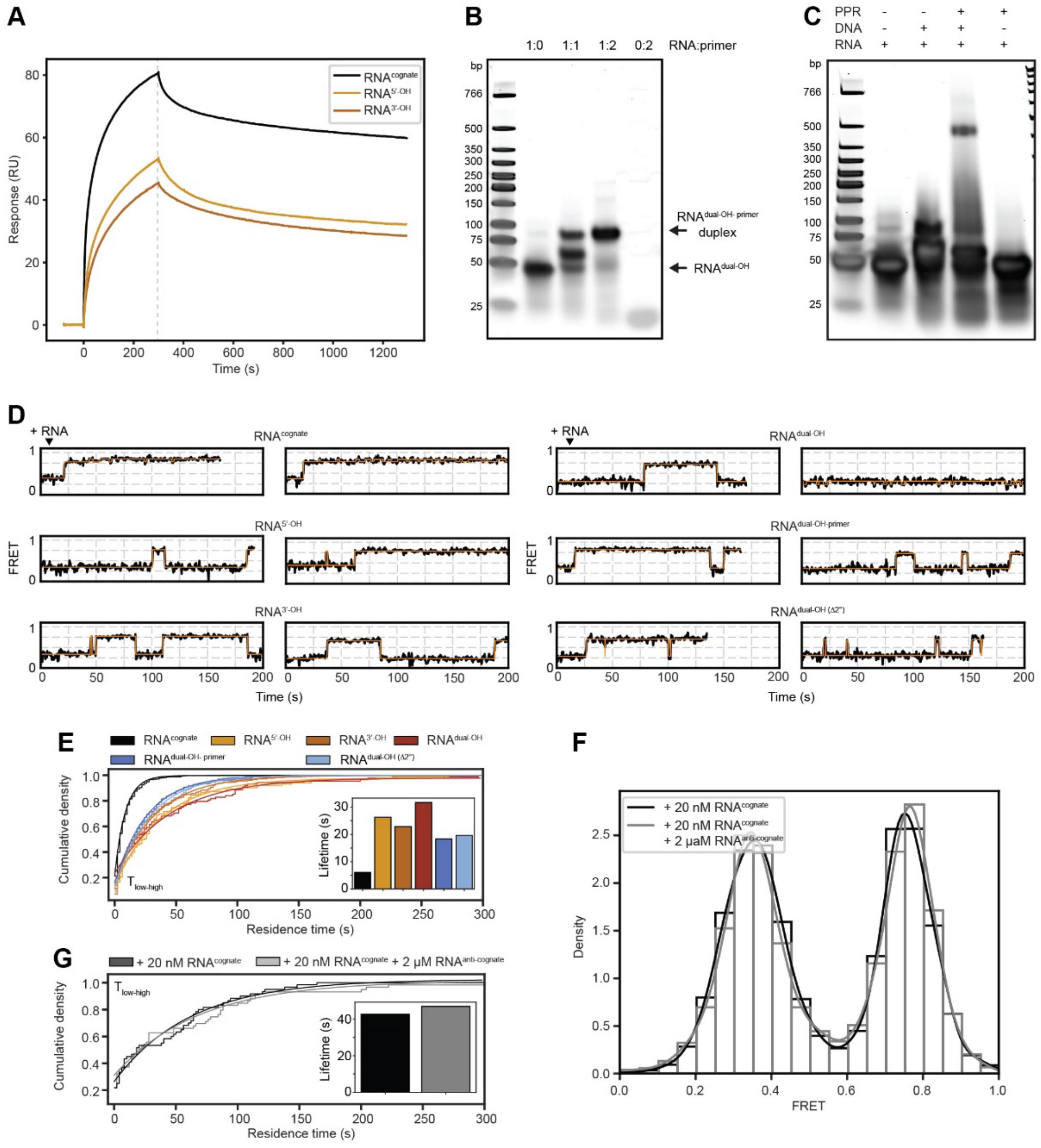

A
Response (RU)
Time (s)
RNA^cognate
RNA^5'-OH
RNA^3'-OH
B
1:0 1:1 1:2 0:2 RNA:primer
bp
766
500
350
300
250
200
150
100
75
50
25
RNA^dual-OH-primer duplex
RNA^dual-OH
C
PPR
DNA
RNA
D
+ RNA
RNA^cognate
RNA^dual-OH
RNA^5'-OH
RNA^dual-OH-primer
RNA^3'-OH
RNA^dual-OH (Δ2")
FRET
Time (s)
E
RNA^cognate RNA^5'-OH RNA^3'-OH RNA^dual-OH
RNA^dual-OH-primer RNA^dual-OH (Δ2")
Cumulative density
T low-high
Lifetime (s)
Residence time (s)
F
+ 20 nM RNA^cognate
+ 20 nM RNA^cognate + 2 μM RNA^anti-cognate
Density
FRET
G
+ 20 nM RNA^cognate
+ 20 nM RNA^cognate + 2 μM RNA^anti-cognate
Cumulative density
T low-high
Lifetime (s)
Residence time (s)

Figure S7: RNA secondary structure, oligonnucleotide length and the presence of flanking non-target sequences delays the binding of cognate ssRNA to dPPR10. (A) Surface plasmon resonance (SPR) traces of the indicated ssRNA constructs (1 µM) binding to immobilized dPPR10. Association of ssRNA to dPPR10 is initiated at t = 0 and proceeds for 300 s. Dissociation curves are generated following removal of ssRNA from solution. **(B)** Native SDS-PAGE gel of $RNA^{dual\text{-}OH}$ (20 µM) following PCR melting and annealing in the absence or presence of DNA primer (20 or 40 µM, 1:1 or 1:2 molar ratio). Quick-load low molecular weight markers (in bp) are shown. **(C)** Native SDS-PAGE gel of $RNA^{dual\text{-}OH}$ (1 µM) that had been incubated for 1 h at 4°C in the absence or presence of dPPR10 (1 µM) supplemented with or without DNA primer (2 µM). The combination of each component within each reaction is indicated above the lane. (**D**) Representative FRET trajectories of dPPR10 upon the injection of the indicated ssRNA (1 µM) after 10 s (indicated above the traces). **(E)** Cumulative histogram of of $T_{low\text{-}high}$ residence times of dPPR10 incubated with the indicated ssRNA. Data is fit with a one-phase association curve and the lifetimes calculated from the fit are shown. **(F)** FRET histogram of dPPR10 incubated in the presence of $RNA^{cognate}$ (20 nM) supplemented with or without a 100-fold excess of $RNA^{anti\text{-}cognate}$ (2 µM). Data is collated from at least 425 individual molecules. **(G)** Cumulative histogram of $T_{low\text{-}high}$ residence times from treatments in which dPPR10 was incubated with $RNA^{cognate}$ (20 nM) in the absence or presence of $RNA^{anti\text{-}cognate}$ (2 µM). Data is fit with a one-phase association curve and the lifetimes calculated from the fit are shown.

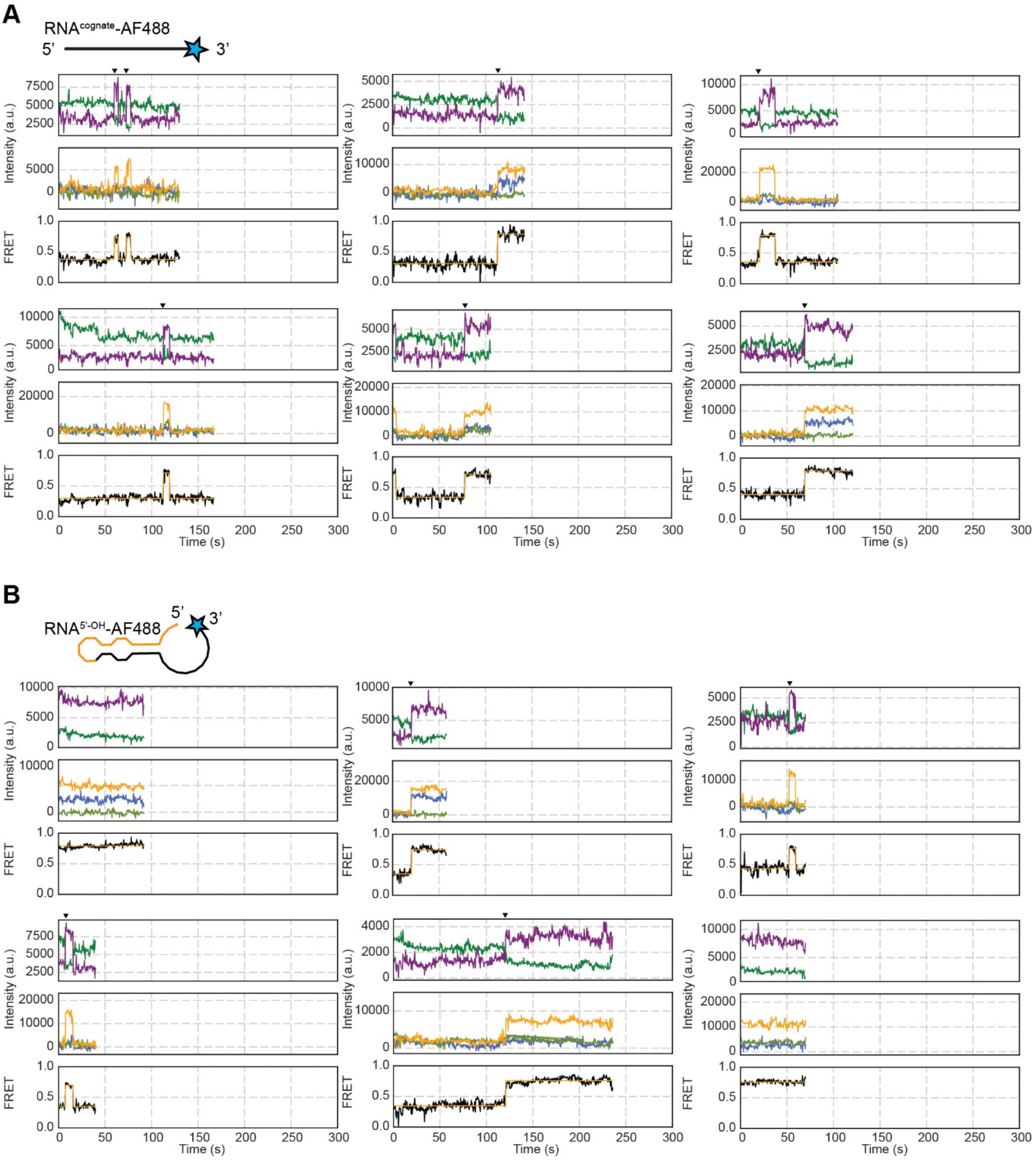

Figure S8: Representative fluorescence intensity and FRET^{PPR} trajectories from 3-colour smFRET experiments. dPPR10 was incubated in the presence of AF488-labelled **(A)** RNA^{cognate} (20 nM) or **(B)** RNA$^{5'\text{-OH}}$ (20 nM) and the fluorescence from AF488, Cy3 and AF647 fluorophores was measured when excited with the 532 nm laser (*top*) or the 488 nm laser (*middle*). FRET^{PPR} trajectories upon excitation with the 532 nm laser (*bottom*) is also shown. Arrows indicate where increases in FRET^{PPR} are correlated with an increase in fluorescence following excitation at 488 nm.

Supplementary Tables

Table S1: Complete protein sequences used in this work. Yellow and pink denotes the position of the AviTag sequence and the position of cysteines for labelling, respectively.

dPPR10 full sequence
MSDKIIHLTDDSFDTDVLKADGAILVDFWAEWCGPCKMIAPILDEIADEYQGKLTVAKLNIDQNPGTAPKYG<br>IRGIPTLLLFKNGEVAATKVGALSKGQLKEFLDANLAGSGSGHMHHHHHHSSGENLYFQGAMGNDVVTYTTL<br>IDGLAKAGRLEEALQLFQEMKEKGVKPDVVTYNTLIDGLAKAGRLEEALQLFQEMKEKGVKPDVVTYTTLID<br>GLAKAGRLEEALQLFQEMKEKGVKPNVVTYNTLIDGLAKAGRLEEALQLFQEMKEKGVKPDVVTYNTLIDGL<br>AKAGRLEEALQLFQEMKEKGVKPSVVTYNTLIDGLAKAGRLEEALQLFQEMKEKGVKPSVVTYNTLIDGLAK<br>AGRLEEALQLFQEMKEKGVKPDVVTYNTLIDGLAKAGRLEEALQLFQEMKEKGVKPDVVTYTTLIDGLAKAG<br>RLEEALQLFQEMKEKGVKPNVVTYTTLIDGLAKAGRLEEALQLFQEMKEKGVKPNVVTYNTLIDGLAKAGRL<br>EEALQLFQEMKEKGVKPSVVTYNTLIDGLAKAGRLEEALQLFQEMKEKGVKPSVVTYTTLIDGLAKAGRLEE<br>ALQLFQEMKEKGVKPNVVTYNTLIDGLAKAGRLEEALQLFQEMKEKGVKPDVVTYNTLIDGLAKAGRLEEAL<br>QLFQEMKEKGVKPDVVTYNTLIDGLAKAGRLEEALQLFQEMKEKGVKPDVVTYNTLIDGLAKAGRLEEALQL<br>FQEMKEKGVKPSVVTNNTLKDGASKAG*
Avi-dPPR10-C2 full sequence
MSDKIIHLTDDSFDTDVLKADGAILVDFWAEWCGPCKMIAPILDEIADEYQGKLTVAKLNIDQNPGTAPKYG<br>IRGIPTLLLFKNGEVAATKVGALSKGQLKEFLDANLAGSGSGHMHHHHHHSSGENLYFQGGSGLNDIFEAQK<br>IEWHEAMGNDVVTYTTLIDGLAKAGRLEEALQLFQEMKEKGVKPDVVTYNTLIDGLAKAGRLEEALQLFQEM<br>KEKGVKPDVVTYTTLIDGLAKAGRLEEALCLFQEMKEKGVKPNVVTYNTLIDGLAKAGRLEEALQLFQEMKE<br>KGVKPDVVTYNTLIDGLAKAGRLEEALQLFQEMKEKGVKPSVVTYNTLIDGLAKAGRLEEALQLFQEMKEKG<br>VKPSVVTYNTLIDGLAKAGRLEEALQLFQEMKEKGVKPDVVTYNTLIDGLAKAGRLEEALQLFQEMKEKGVK<br>PDVVTYTTLIDGLAKAGRLEEALQLFQEMKEKGVKPNVVTYTTLIDGLAKAGRLEEALQLFQEMKEKGVKPN<br>VVTYNTLIDGLAKAGRLEEALCLFQEMKEKGVKPSVVTYNTLIDGLAKAGRLEEALQLFQEMKEKGVKPSVV<br>TYTTLIDGLAKAGRLEEALQLFQEMKEKGVKPNVVTYNTLIDGLAKAGRLEEALQLFQEMKEKGVKPDVVTY<br>NTLIDGLAKAGRLEEALQLFQEMKEKGVKPDVVTYNTLIDGLAKAGRLEEALQLFQEMKEKGVKPDVVTYNT<br>LIDGLAKAGRLEEALQLFQEMKEKGVKPSVVTNNTLKDGASKAG*

Table S2: Crystallographic data and refinement statistics. Numbers in parenthesis correspond to the highest resolution shell.

**Data collection**	
**Space group**	*P*1
**Unit cell dimensions**	
**a, b, c (Å)**	43.34, 51.75, 51.97
**α, β, γ (°)**	118.1, 97.2, 96.0
**Wavelength (Å)**	0.9537
**Resolution (Å)**	44.92-2.01 (2.06-2.01)
**R_{merge} (%)**	5.1 (22.8)
I/σI	9.0 (2.1)
**$CC_{1/2}$**	0.995 (0.786)
**Completeness (%)**	97 (90.8)
**Redundancy**	2.0 (1.6)
**Wilson B ($Å^2$)**	26.9
**Refinement**	
**Resolution (Å)**	44.92 – 2.00
**No. reflections**	23919
**R_{work}/R_{free}**	18.2/24.7
**No. Atoms**	10755
**Protein**	9773
**RNA**	768
**Water**	214
**Average B-factor ($Å^2$)**	32.2
**R.m.s deviations:**	
**Bond lengths (Å)**	0.014
**Bond angles (°)**	1.6
**Ramachandran analysis**	
**Favored (%)**	96.0
**Allowed (%)**	4.0
**Outliers (%)**	0.0
